# Measuring and optimizing the urban community resilience against public health emergencies: a case study in Nanjing, China

**DOI:** 10.3389/fpubh.2025.1691666

**Published:** 2025-10-16

**Authors:** Peng Cui, Saiya Cao, Ruize Qin, Fan Zhang, Dezhi Li, Lan Feng

**Affiliations:** ^1^School of Civil Engineering, Nanjing Forestry University, Nanjing, China; ^2^Department of Applied Physics and Electronics, Umeå Universitet, Umeå, Sweden; ^3^A. James Clark School of Engineering, University of Maryland, College Park, MD, United States; ^4^Department of Building and Real Estate, The Hong Kong Polytechnic University, Kowloon, Hong Kong SAR, China; ^5^Department of Construction and Real Estate, Southeast University, Nanjing, China

**Keywords:** urban community resilience, public health emergencies, Bayesian network, resilience metrics, optimization strategies

## Abstract

**Introduction:**

Urban communities, as the basic unit of urban governance, play a crucial role in responding to public health emergencies (PHEs). This study aims to investigate the resilience measurement and optimization strategies of urban communities in responding to PHEs in order to improve their resilience.

**Methods:**

The study constructed a resilience assessment framework and identified 31 key influencing factors to measure the resilience of case communities in Nanjing. Through sensitivity analysis, static optimization strategies were proposed from social, environmental, and economic levels. Dynamic Bayesian network inference simulation and importance analysis were used to propose dynamic optimization strategies from pre, during, and long-term perspectives.

**Results:**

Through the combination of dynamic and static strategies, community managers promote resilience building from both short-term and long-term perspectives.

**Discussion:**

The study provides a valuable reference for comprehensively improving the emergency management system.

## 1 Introduction

As cities continue to expand, they are increasingly vulnerable to a range of uncertainties originating from both external and internal sources (1). In addition to common natural disasters such as earthquakes, typhoons, and floods, the severe consequences of public health emergencies (PHEs) should not be underestimated. Since the implementation of the International Health Regulations in 2007, the WHO has declared seven global PHEs, including outbreaks of H1N1 influenza, Ebola virus, Zika virus, and monkeypox. Although PHEs occur infrequently, they have a broad-reaching impact and spread rapidly, posing substantial threats to human health and socio-economic stability. The global outbreak of the COVID-19 pandemic in 2020 has had profound effects on global economic development. According to World Health Organization statistics, as of August 2024, the cumulative number of globally diagnosed cases stands at 776 million, with approximately 7.06 million confirmed deaths. This public health crisis has had a profound impact on global economic development (2), leading to significant deterioration in the economic conditions of sectors such as manufacturing, agriculture, the food industry, education, sports, and entertainment (3).

The COVID-19 pandemic has presented substantial challenges to existing urban response systems for PHEs, spanning from pre-disaster prevention to mid-disaster management. Communities, as fundamental units of human activity and foundational elements of urban governance, play a pivotal role in the early stages of emergency response and are critical for the development of comprehensive public health service systems. During the pandemic, stringent measures at the community level, such as community blockades (4, 5) and strict restrictions on entry and exit (6), were effective in preventing the spread of the outbreak, controlling its transmission, and reducing the loss of life and property (7).

In the face of unpredictable and interconnected public health crises, post-disaster mitigation tends to be reactive, allowing for only limited reductions in losses once a disaster has occurred. The most effective approach to disaster risk reduction involves fostering communities that can self-organize and self-adjust to evolving emergencies, thereby achieving optimization and stability—essentially embedding urban community resilience. The concept of “resilience,” encompassing elasticity, resistance, and adaptability, originated in engineering before being widely adopted across various disciplines, including ecology (8), psychology (9), economics (10), and safety management (11). In the context of disaster risk, resilience is defined as “the ability of a system or its components to absorb disturbance and still retain its essential functions following a disaster event” (12). The process of resilience in mitigating disasters and reducing damage can be divided into four stages: prevention and preparation, absorption, rapid recovery, and adaptation and transformation. In this study, urban community resilience refers to the capacity of community systems to resist disruptions caused by uncertainty, while also recovering and adapting as necessary (13).

Since the Second United Nations World Conference on Disaster Risk Reduction, governments, institutions, and academic communities in countries such as the United States, Germany, Japan, and others have been exploring the development of resilient communities. Identifying appropriate dimensions to analyze the factors influencing resilience and establishing standardized and effective metrics for measuring resilience are essential steps in assessing the progress of resilience-building efforts within communities. To evaluate urban community resilience, scholars have employed various frameworks, including the 4R Theory (14), the Disaster Resilience Local Framework Model (DROP) (15), the Community Climate Resilience Assessment (CCRAM) (16), the Interpretive Structural Modeling - Analytic Hierarchy Process (ISM-AHP) model (17), the Community Disaster Resilience Framework (CDRF) (18), and the iRe-CoDeS Framework (19). These frameworks incorporate dimensions such as spatial resilience, capital resilience, social resilience, and governance resilience (20–22) to quantify the level of urban community resilience.

In recent years, scholars have progressively incorporated the concept of resilience into the prevention, control, and comprehensive management of communities during PHEs. This paradigm not only leverages the inherent advantages of communities in crisis situations but also introduces innovative strategies for preventing and managing public health challenges in non-crisis periods. Research has emphasized the importance of efficient collaboration and coordinated prevention and control efforts among community stakeholders. Findings suggest that critical factors such as government policy prioritization and tiered guidance (23), neighborhood social capital (6), resource allocation (24), and the active engagement of community residents and volunteer organizations (6) significantly enhance the effectiveness of community-based prevention and control measures (23, 25). Additionally, several scholars have proposed frameworks for prevention and control planning informed by resilience theory (7, 26–28). Research on urban community resilience in the context of PHEs predominantly focuses on collaborative governance, resident participation, external interventions, and the development of organizational systems (29). However, there is limited exploration of urban community resilience as a sociological construct from an overarching network perspective, incorporating essential elements such as resources, the environment, and activities. Consequently, integrating resilience theory into the framework of urban community prevention and control during PHEs—by identifying critical factor nodes, clarifying the hierarchical relationships among resilience factors, and modeling the likelihood of resilience actions—is paramount for enhancing communities’ capacity to proactively adapt to emergencies and safeguarding vulnerable populations at the grassroots level.

Building upon identified research, this study aims to achieve the following objectives: First, a novel framework is proposed to assess urban community resilience for PHEs, incorporating key influencing factors across three dimensions: social, environmental, and economic. Second, leveraging Bayesian networks, an optimization strategy is introduced to enhance urban community resilience in addressing future public health challenges. This study not only offers actionable strategies for community administrators to implement resilience-building initiatives but also provides theoretical foundations to improve the effectiveness of PHEs management systems.

## 2 Methods

### 2.1 Research idea

This study aims to investigate the application of urban community resilience for PHEs using an “Identification-Measurement-Optimization” approach. The methodology follows these steps: (1) Through a comprehensive literature review, this study identifies influencing factors within the resilience framework. Identify the influencing factors within the frameworks of social, environmental, and economic resilience through a comprehensive literature review; (2) Apply the Decision-Making Trial and Evaluation Laboratory (DEMATEL) method to assess the relationships between these factors through matrix transformation and threshold filtering; (3) Develop a Bayesian network-based model to measure urban community resilience for PHEs, using communities in Nanjing, China, as a case study; (4) Measure resilience levels and diagnose the key factor chains that lead to resilience failure through reverse reasoning; (5) Generate static optimization strategies for urban community resilience for PHEs based on sensitivity analysis of the influencing factors; (6) Design simulation scenarios to analyze changes in community resilience values and importance indicators over time, subsequently generating dynamic optimization strategies for urban community resilience for PHEs, thereby maximizing the role of community resilience during emergencies.

### 2.2 Establishment of the Bayesian network

Given the numerous uncertainties associated with PHEs, Bayesian Network (BN) can quantify these uncertainties through probabilistic reasoning and represent the causal relationships between factors (30). Accordingly, this study chooses to construct a BN model to assess urban community resilience for PHEs. Initially, influencing factors are defined as network nodes, and based on their internal hierarchical relationships, the paths of influence are delineated to construct a resilient network structure. Subsequently, expert assessments regarding the state distribution of influencing factors are obtained through surveys. Next, fuzzy comprehensive evaluation is employed to calculate the prior probabilities of root nodes, and the Leaky Noisy-OR model is introduced to determine the conditional probability tables for non-root nodes. Finally, data are imported into NETICA software to perform forward and backward inference diagnostics on the BN of urban community resilience for PHEs.

#### 2.2.1 Determination of the relationship between influencing factors

This study employs the DEMATEL method to identify causal relationships among nodes and to delineate the structure of the BN for urban community resilience for PHEs. The BN is represented as a directed acyclic graph illustrating the interrelationships among influencing factors. The DEMATEL method has been extensively applied in disaster management research (31–33) and is particularly effective in analyzing the factors that influence community resilience during PHEs. The relationships between these factors are established through a structured survey, which is conducted as follows:

1. Form a direct impact matrix

The survey scores are summarized, averaged, and then rounded to form the direct influence matrix *A* = [*a_ij_*]*_n × n_*, where *a_ij_* represents the degree of influence that factor *xi* has on factor *X_j_*, with *a_ij_* = *0* when *i* = *j*.

2. Normalizing the Direct Influence Matrix

To standardize the dimensions, the maximum value normalization method is applied. The sum of each row in matrix *A* is calculated, and the maximum value is identified. Using this value, the standardized matrix *B* = [*b_ij_*]*_n × n_* is derived according to Equation 1, where *b_ij_* ranges between 0 and 1.


(1) $$B=\frac{a_{\mathrm{ij}}}{\max\sum_{j=1}^{n}a_{\mathrm{ij}}}$$

3. Solving the Comprehensive Influence Matrix

The community functions as a micro-urban unit comprised of multiple entities and elements, characterized by complex and diverse network relationships. The interconnections among urban community resilience factors are multifaceted. Matrix *A* captures the direct influences between these factors, while successive multiplications of matrix *B* represent the growing indirect influences among elements. This process culminates in the comprehensive influence matrix *T*, as shown in Equation 2:


(2) $$T=B{\left(I-B\right)}^{-1}$$

4. Calculation of Four Metric Indices

Based on matrix *T*, four metric indices for influencing factors of urban community resilience for PHEs are derived. The influence degree *f_i_* is calculated as the sum of each row in matrix *T*, representing the overall impact of each factor on others, as shown in Equation 3:


(3) $$\begin{array}{c} f_{i}=\sum_{j=1}^{n}t_{\mathrm{ij},}(i=1,2,\ldots ,n)\\ f_{i}=\sum_{j=1}^{n}t_{\mathrm{ji}}(i=1,2,\ldots ,n) \end{array}$$

The affected degree *e_i_* is the sum of each column in matrix *T*, indicating the overall influence received by each factor from others, as shown in Equation 4:


(4) $$\begin{array}{c} \;\\ \begin{array}{c} e_{i}=\sum_{j=1}^{n}t_{\mathrm{ij},}(i=1,2,\ldots ,n)\\ e_{i}=\sum_{j=1}^{n}t_{\mathrm{ji}}(i=1,2,\ldots ,n) \end{array} \end{array}$$

The centrality *m_i_* is calculated as the sum of *f_i_* and *e_i_*, reflecting the overall significance of the factor within the evaluation system, as shown in Equation 5:


(5) $$m_{i}=f_{i}+e_{i}$$

The causality degree *n_i_* is defined as the difference between *f_i_* and *e_i_*. If *n_i_* > 0 indicates that the factor exerts a greater influence on others, serving as a causal factor for resilience. Conversely, *n_i_* < 0 suggests that the factor is more influenced by others, functioning as an effect factor.


(6) $$n_{i}=f_{i}-e_{i}$$

#### 2.2.2 Calculating the prior probabilities of root nodes

The prior probability values of the root nodes in the BN are derived through expert questionnaires combined with the fuzzy comprehensive evaluation method, representing the failure probabilities of the corresponding influencing factors. In accordance with Wickens’ Signal Detection Theory, the failure probabilities of the variable factors are classified into seven levels using fuzzy linguistic terms. The specific trapezoidal fuzzy numbers and the corresponding evaluation levels are presented in Table 1.

**Table 1:** Forms of fuzzy numbers.

Level	Fuzzy linguistic term	Fuzzy number
1	Very Low (VL)	(0, 0, 0.1, 0.2)
2	Low (L)	(0.1, 0.2, 0.2, 0.3)
3	Fairly Low (FL)	(0.2, 0.3, 0.4, 0.5)
4	Medium (M)	(0.4, 0.5, 0.5, 0.6)
5	Fairly High (FH)	(0.5, 0.6, 0.7, 0.8)
6	High (H)	(0.7, 0.8, 0.8, 0.9)
7	Very High (VH)	(0.8, 0.9, 1, 1)

Due to variations in the understanding and experience of experts in urban community resilience and emergency management, the arithmetic mean method is insufficient for addressing these discrepancies and obtaining accurate results. This study employs the weighted average method to process fuzzy ratings, comparing each expert’s evaluation with the mean value and assigning distinct weight coefficients to each expert. The weight assigned to an expert is proportional to the proximity of their rating to the mean, with experts whose evaluations are closer to the mean receiving higher weights. Suppose the fuzzy rating of a particular expert is $F_{k}$ = (*F_k1_, F_k2_, F_k3_, F_k4_*) where *k* = *1, 2, …, n.*

1. Calculating the Arithmetic Mean of Trapezoidal Fuzzy Numbers $F_{a}$ , from *n* Experts, as shown in Equation 7:

(7) $$F_{a}=(F_{a1},F_{a2},F_{a3},F_{a4})=(\begin{array}{l} \frac{1}{n}\sum_{k=1}^{n}F_{k1},\frac{1}{n}\sum_{k=1}^{n}F_{k2},\\ \frac{1}{n}\sum_{k=1}^{n}F_{k3},\frac{1}{n}\sum_{k=1}^{n}F_{k4} \end{array})$$

2. Calculating the Distance Between $F_{k}$ and $F_{a}$ , as shown in Equation 8:

(8) $$d(F_{k},F_{a})=\frac{1}{4}(|F_{k1}-F_{a1}|+|F_{k2}-F_{a2}|+|F_{k3}-F_{a3}|+|F_{k4}-F_{a4}|)$$

3. Calculating the Similarity Between $F_{k}$ and $F_{a}$ , as shown in Equation 9:

(9) $$S(F_{k},F_{a})=1-\frac{d(F_{k},F_{a})}{\sum_{k=1}^{n}d(F_{k},F_{a})}$$

4. Calculating the Expert Evaluation Weight $W_{k}$ , as shown in Equation 10:

(10) $$W_{k}=\frac{S(F_{k},F_{a})}{\sum_{k=1}^{n}S(F_{k},F_{a})}$$

5. Calculating the Weighted Comprehensive Fuzzy Evaluation Number *F*, as shown in Equation 11:

(11) $$F=(F_{1},F_{2},F_{3},F_{4})=\sum_{k=1}^{n}W_{k}F_{k}=\sum_{k=1}^{n}(\frac{S(F_{k},F_{a})}{\sum_{k=1}^{n}S(F_{k},F_{a})}F_{k})$$

6. Calculating the Weighted Aggregated Fuzzy Evaluation Value, as shown in Equation 12:

(12) $$P=\frac{F_{1}+F_{2}+F_{3}+F_{4}}{4}$$

#### 2.2.3 Calculating the conditional probabilities of non-root nodes

(1) The Noisy-OR model is a commonly used simplification tool in BN to represent the relationship between *n* mutually independent parent nodes *Z_1_*, *Z_2_*, …, *Z_n_* and a child node *Y*. All nodes are binary variables, with only two states: “0” representing no failure and “1” representing failure. Additionally, the Noisy-OR model assumes that the outcome of *Y* depends solely on the values of *X_i_* (*i* = 1, 2, …, *n*) and is independent of other variables. In other words, there is a failure connection probability *P_i_* (*i* = 1, 2, …, *n*) between each *x_i_* and *Y* which satisfies Equation 13:


(13) $$P_{i}=(Y|X_{i})=(Y|\bar{X_{1},}\bar{X_{2},}\ldots ,X_{i},\ldots ,\bar{X_{n}})$$

It represents the probability that *Y* fails given that only *x_i_* fails. In other words, if *Y* = 1 (failure), then at least one of the *x_i_* must be 1 (failure). Assuming that all parent nodes with *x_i_* = 1 (failure) form the set *X_T_*, the conditional probability of *Y* failing is calculated as shown in Equation 14:


(14) $$P=1-\prod_{i:X_{i}\in X_{T}}(1-P_{i})$$

Although the Noisy-OR model cannot capture complex interactions or non-monotonic relationships, it is often used to explain and analyze causal relationships in practical problems due to its simplicity, intuitiveness, and computational efficiency. However, the BN for urban community resilience may not encompass all influencing factors of PHEs, which undermines the assumption in the Noisy-OR model that “the failure of a child node is independent of variables other than its parent nodes.” Although these potential variables are not identified or selected as parent nodes, they can still introduce biases into the results. The Leaky Noisy-OR model can compensate for this shortcoming by introducing a default node *Z_L_* to represent other potential variables that are not considered but may still cause the child node to fail.

Even if all parent nodes *Z_i_* do not fail, the child node *Y* may still fail due to the failure of the default node *Z_L_*. Let *P_L_* denote the probability that the child node *Y* fails when only the default node *Z_L_* fails and all other parent nodes do not, with the confidence interval in this study set to 0.9 (34), i.e., *P_L_* = 0.1. A 90% confidence level indicates that, if the sampling process were repeated numerous times, approximately 90% of the resulting confidence intervals would encompass the true parameter value. This approach balances the need for a high degree of reliability with the provision of more precise estimates, thereby mitigating the risk of being excessively conservative. Assume that the set of all failed parent nodes is *Z_T_*, and *Pi* represents the probability that the child node *Y* fails when only parent node *Z_i_* fails while all other parent nodes do not. This probability is primarily obtained using the same method as for root nodes. The conditional probability of failure for each non-root node can then be calculated using Equation 15.


(15) $$P=1-(1-P_{L})\prod_{i:Z_{i}\in Z_{T}}\frac{\left(1-P_{i}\right)}{\left(1-P_{L}\right)}$$

## 3 Results

### 3.1 Identification of influencing factors of urban community resilience for PHEs

#### 3.1.1 Theoretical framework for assessing urban community resilience

PHEs are defined by their abrupt onset and inherent uncertainty. Therefore, the assessment of urban community resilience must account for these dynamic changes. The Pressure-State-Response (PSR) model serves as a framework to analyze the relationship between environmental pressures, system status, and policy responses. This framework aligns closely with the dynamic processes urban communities undergo in the face of PHEs. During such incidents, communities initially face external pressures, which prompt changes in their status. In response, communities implement various measures to mitigate the pressure and restore stability. The PSR model offers a robust theoretical framework for understanding the “pressure-status-response” dynamic cycle, facilitating a comprehensive understanding of the formation and evolution of urban community resilience in urban settings.

The Social-Economic-Natural Composite Ecosystem (SENCE) theory conceptualizes society, economy, and nature as an interconnected composite system, emphasizing their interdependence and mutual influence. As a complex system, the resilience of urban communities is shaped by a range of interrelated factors, including the characteristics of community residents, social capital, material resources, financial assets, the natural environment, and infrastructure. The SENCE theory integrates these three dimensions, offering a systematic framework for analyzing the multi-level impacts on urban community resilience, thereby overcoming the limitations of unidimensional analyses.

In conclusion, the PSR model centers on the causal chain of “pressure - status - response,” emphasizing the logical relationship between external shocks and system responses. The SENCE theory enhances the understanding of the “status” dimension by revealing the intricate interactions among the social, economic, and environmental subsystems. Consequently, this study integrates the PSR model with the SENCE theory, combining the two to form a more comprehensive framework for resilience analysis. The dimensions of pressure, status, and response align with the pre-disaster, during-disaster, and post-disaster phases, respectively, facilitating resilience evaluation across these distinct stages. In accordance with the SENCE theory, the factors influencing urban community resilience are classified into social, economic, and environmental dimensions. The social dimension addresses the personal characteristics of community residents and factors related to community management. The economic dimension encompasses the community’s available financial resources and its capacity for effective resource allocation. The environmental dimension includes a wide array of environmental factors from a public health perspective, such as natural conditions, built environments, and physical infrastructure within the community.

**Figure 1:**
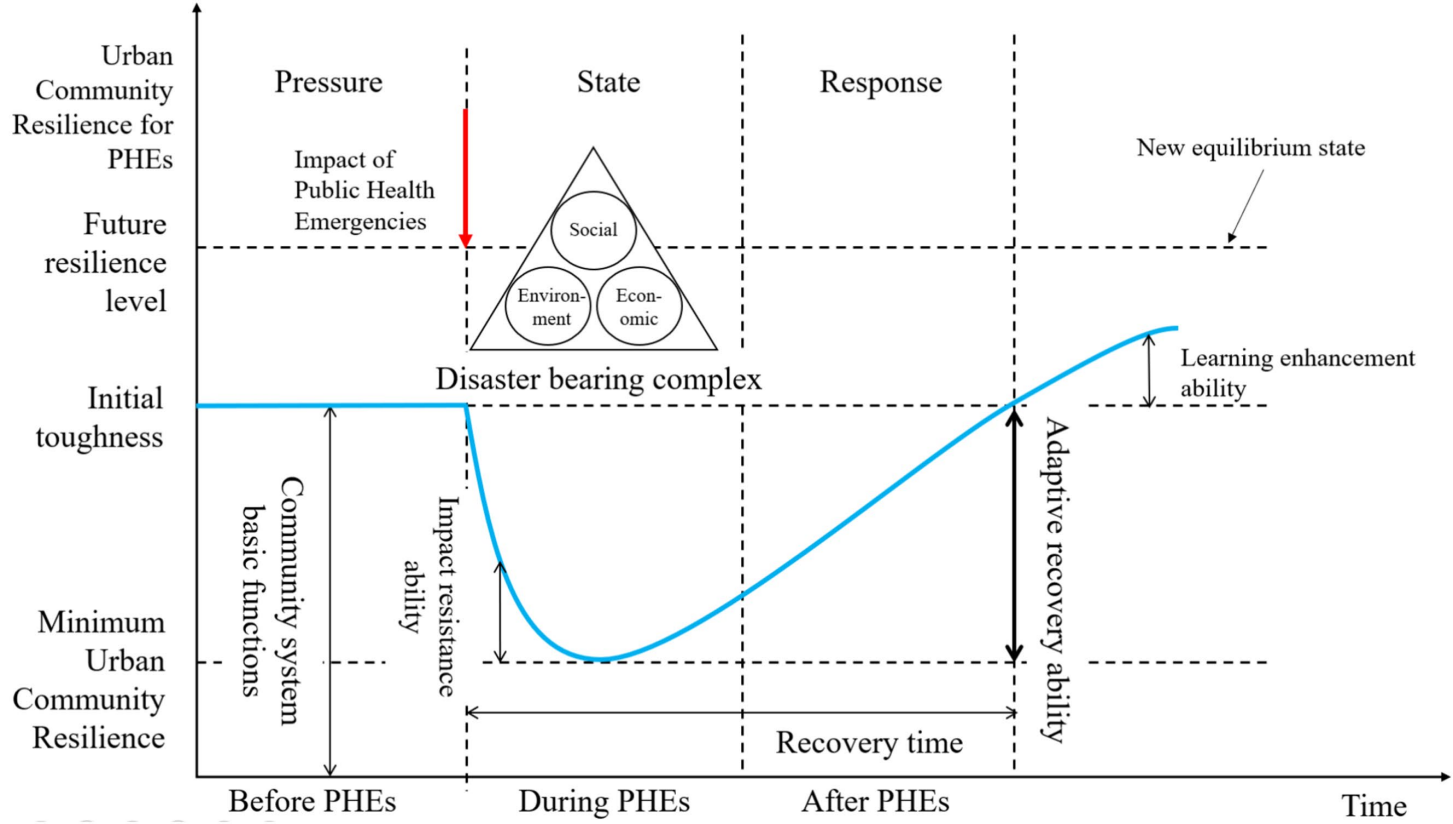
Urban community resilience for PHEs framework curve.

The urban community resilience model for PHEs can be categorized into three dimensions: (1) Pressure: This dimension refers to the initial resilience level of the community when exposed to potential pressures from PHEs, specifically its capacity to withstand hazardous factors and risk-prone environments. (2) State: This dimension reflects the capacity of the urban community complex system during PHEs, characterized by robustness, redundancy, and other key features of the community’s inherent resources. (3) Response: This dimension relates to the community’s ability to implement measures and adapt to recovery across social, economic, and environmental systems under the influence of PHEs. It is defined by the strategic and timely response capabilities that characterize resilience. The specific framework curve is illustrated in Figure 1.

This study focuses on the factors influencing resilience as the core domain, proposing that the three primary categories—social, environmental, and economic factors—collectively shape resilience. These factors interact and exert mutual influence, creating a synergistic and interconnected mechanism driven by their inherent relational logic. This interaction culminates in the development of a theoretical model of the factors influencing resilience.

#### 3.1.2 Identification of influencing factors

To comprehensively identify the influencing factors of resilience, this study conducted a search on the Web of Science using the search string TS = [(“urban resilience” OR “city resilience”) AND (“epidemic” OR “COVID-19” OR “Public health emergency” OR “pandemic”)]. This search yielded the identification of 31 influencing factors, as illustrated in Table 2.

**Table 2:** Framework of influencing factors for urban community resilience for PHEs.

Resilience characterization	Dimension	Influencing factor layer	References
Social resilience (Soc)	Pressure (P)	Level of PHEs (SocP1)	Scherzer et al. (39), Zhang et al. (6)
		Vulnerable groups (SocP2)	
	State (S)	Population structure (SocS1)	Cui et al. (40), Deng et al. (41), Kais and Islam (42), Liu et al. (43), Niu et al. (44), Wang et al. (45), Yan et al. (46), Zhao et al. (47)
		Resident health status (SocS2)	
		Resident educational level (SocS3)	
		Risk awareness (SocS4)	
		Rules and regulations (SocS5)	
		Publicity and education (SocS6)	
	Response (R)	Resident belongingness (SocR1)	Liu et al. (48), Niu et al. (44), Pfefferbaum et al. (49), Reveilhac (50), Shi et al. (22), Wang et al. (51), Yan et al. (46), Zhao et al. (47)
		Public service (SocR2)	
		Public participation (SocR3)	
		Social network relationships (SocR4)	
		Community emergency management capability (SocR5)	
		Past experience (SocR6)	
		Government leadership functions (SocR7)	
Environmental Resilience (Env)	State (S)	Sanitation state (EnvS1)	Chen et al. (52), Liu et al. (53), Niu et al. (44), Shi et al. (22), Su et al. (54)
		Community quality (EnvS2)	
		Public space (EnvS3)	
		Entrance/exit management (EnvS4)	
	Response (R)	Accessibility of medical facilities (EnvR1)	Deng et al. (41), Jiang et al. (55), Li et al. (56), Niu et al. (44), Scherzer et al. (39), Summers et al. (57), Zhao et al. (47)
		Emergency shelter (EnvR2)	
		Living supporting facilities (EnvR3)	
		Transportation robustness (EnvR4)	
Economic Resilience (Eco)	State (S)	Resident employment (EcoS1)	Deng et al. (41), Niu et al. (44), Shi et al. (22), Summers et al. (57), Zhao et al. (47)
		Resident income (EcoS2)	
		Social insurance (EcoS3)	
		Community assets (EcoS4)	
	Response (R)	Medical supplies (EcoR1)	Niu et al. (44), Su et al. (54), Summers et al. (57), Wang et al. (51), Zhao et al. (47)
		Capital investment (EcoR2)	
		Communication system (EcoR3)	
		Intelligent supervision (EcoR4)	

### 3.2 Constructing a Bayesian network for urban community resilience for PHEs

#### 3.2.1 Influencing factor analysis based on DEMATEL

Among the three subsystems of urban community resilience for PHEs, the environmental and economic dimensions exhibit fewer influencing factors and a flatter hierarchical structure. In contrast, the social dimension involves a greater number of influencing factors with more complex internal logical relationships. Therefore, this study focuses specifically on analyzing the causal relationships among the influencing factors within the social resilience dimension.

In this study, we selected over 10 communities in the main urban area of Nanjing, all of which had undergone high-risk containment and control measures. Representatives from neighborhood committees, property volunteers, health service centers, and street offices in these communities were invited to participate in offline interviews, and those with extensive experience were selected as interviewees. Subsequently, using the Questionnaire Star platform, an online survey was conducted. The survey invited these representative community staff, as well as experts and scholars in disaster management and urban community resilience, to complete a scoring questionnaire based on their professional knowledge and past work experience. The participants were asked to quantify the influence between each pair of factors using a 0–4 scale, ranging from “no influence” to “low,” “medium,” “high,” and “very high” influence. The scoring form is provided in Appendix 1.

A total of 43 questionnaires were distributed, and 31 valid responses were collected (responses completed in less than 450 s were excluded). The questionnaire scoring data were then processed into matrices, normalized, and aggregated according to the method outlined in Section 2.2.1 to obtain the comprehensive influence matrix *T*. Using Equations 3–6, the four-degree indicators for each social influence factor were calculated, and the summary is shown in Table 3. Preliminary analysis of the causality degree indicator shows that 10 factors with a positive n belong to the cause group, while 5 factors with a negative n belong to the effect group.

**Table 3:** DEMATEL analysis of social influencing factors of urban community resilience for PHEs.

Influencing factors	Influence degree (f)	Affected degree (e)	Centrality (m)	Causality (n)	Factor attribute
SocP1	3.977	4.408	8.385	−0.431	Effect group
SocP2	3.387	3.309	6.696	0.078	Cause group
SocS1	2.100	1.667	3.767	0.433	Cause group
SocS2	2.901	4.184	7.085	−1.283	Effect group
SocS3	3.556	1.529	5.086	2.027	Cause group
SocS4	2.474	4.428	6.903	−1.954	Effect group
SocS5	4.081	3.480	7.561	0.601	Cause group
SocS6	3.794	3.607	7.401	0.187	Cause group
SocR1	3.550	3.242	6.792	0.308	Cause group
SocR2	3.963	3.491	7.454	0.473	Cause group
SocR3	3.537	4.646	8.183	−1.109	Effect group
SocR4	4.530	4.031	8.561	0.500	Cause group
SocR5	3.163	4.764	7.927	−1.601	Effect group
SocR6	3.719	3.196	6.915	0.523	Cause group
SocR7	3.530	2.282	5.812	1.249	Cause group

**Figure 2:**
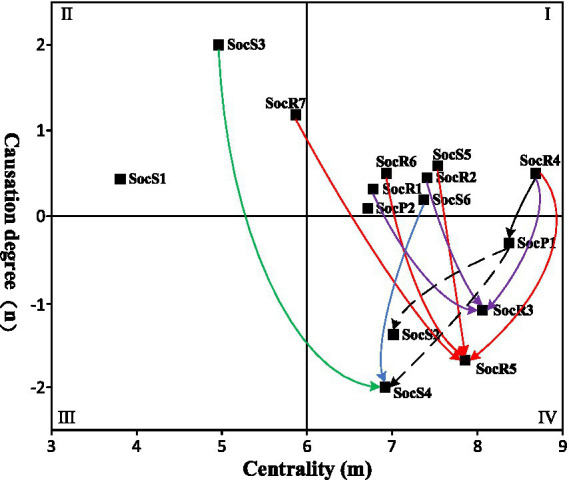
Critical impact path between variables of resilience in social dimension.

The threshold method is employed to reveal the mechanisms of influence among social resilience factors within the community, as illustrated in Figure 2. In this study, drawing on methodological precedents from the literature, we utilized percentile-based threshold determination criteria. The threshold *α* was set at the 95th percentile of all coefficients within the comprehensive impact matrix *T* (35). This statistical boundary demarcation ensures that the assessment results prioritize the identification of operationally significant influence pathways, while effectively filtering out noise from low-impact connections that could otherwise distort model outcomes. Based on the threshold calculation result (0.35746), relationships with a comprehensive influence coefficient exceeding this threshold are identified as key influence pathways. Risk awareness, public participation, social network relationships, and community emergency management capacity each exhibit more than two key pathways, suggesting closer and more significant connections with other factors. Except for the two dashed lines representing influence pathways between the effect factors, all other pathways flow from cause factors to effect factors, aligning with causal logic. Key influence pathways are differentiated by color based on the effect factors they lead to: blue pathways exclusively connect to state-bearing factors, while purple pathways exclusively connect to response and disaster relief factors. One red pathway connects rules and regulations to community emergency management capacity, indicating that the community’s social state-bearing capacity can influence its social response and disaster relief capabilities to some extent, consistent with the internal mechanism of the PSR model. A black pathway from social network relationships to the level of PHEs suggests that community social response and disaster relief can influence social pressures to some extent, aligning with the feedback mechanism of the PSR model.

#### 3.2.2 Bayesian network structure of urban community resilience for PHEs

**Figure 3:**
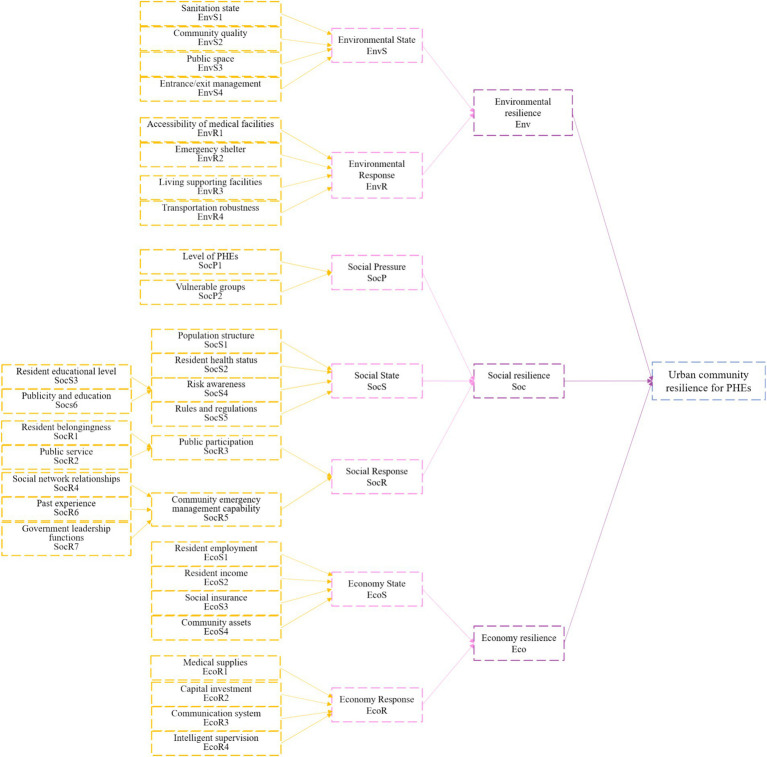
BN structure of urban community resilience for PHEs.

The internal hierarchy of resilience, along with the degree of influence and causal attributes among the influencing factors, has been clarified. Factors with high causality degrees are designated as root nodes in the BN structure, while factors such as risk awareness, public participation, and community emergency management capacity, which exhibit negative causality degrees, are classified as non-root nodes in the BN structure. Finally, key causal pathways within the same dimension are prioritized, with pathways exhibiting higher influence coefficients preferred when factors reside within the same dimension. This methodology ultimately determines the BN structure of resilience, as illustrated in Figure 3.

### 3.3 Case study

#### 3.3.1 Basic information of the case

This study undertook a comprehensive survey of the fundamental characteristics of communities in Nanjing’s urban core, including their founding year, geographic location, and construction scale. Four communities—A, B, C, and D—were selected as case studies, with their profiles provided in Table 4. Each of these communities has distinct characteristics, which allow the urban community resilience BN model developed in this study to demonstrate broad applicability.

**Table 4:** Community basic information.

Community	Area (km^2^)	Total households	Resident population	Population density (persons/km^2^)	Community characteristics
A	0.090	1,827	4,775	53,055	The community has a long history, with relatively outdated facilities. The majority of residents are older adults, consisting primarily of teachers, students, and other intellectuals.
B	0.175	3,520	8,700	49,714	Residents are highly educated, have access to excellent medical care, and the neighborhood fosters a strong sense of community life.
C	0.500	3,000	9,000	18,000	Younger households predominate, the community environment is newer, and the facilities are well-equipped.
D	1.500	6,373	17,000	11,333	The community has a low population density, with a significant influx of non-local residents, and it has experienced pandemic lockdown management.

The survey was conducted online, as detailed in Appendix 2. The respondents included frontline managers from the four selected communities, as well as experts and scholars in the fields of urban community resilience and emergency management. A total of 32 questionnaires were distributed, with 22 valid responses received. The criteria for selecting valid questionnaires were as follows: (1) Respondents had limited knowledge of urban community public health safety issues (e.g., policies, technology, knowledge), categorized as either “almost unaware” or having “little knowledge”; (2) the time taken to complete the questionnaire was less than 600 s; and (3) respondents had less than 1 year of relevant work or research experience.

Among the 22 valid respondents, 81% held a bachelor’s degree or higher, and 75% had at least 5 years of work experience. The respondents’ job positions were distributed as follows: 31% were involved in emergency management, 14% in party and human resources, 18% in healthcare, and 36% held academic positions at universities.

#### 3.3.2 Solving the BN of urban community resilience for PHEs

1. Solving the Prior Probabilities of Root Nodes

Based on the valid questionnaire data, the prior probabilities of the root nodes of BN are calculated using Equations 7–12. The results are summarized in Table 5.

2. Calculation of Conditional Probabilities for Non-root Nodes

**Table 5:** Prior probabilities of root nodes in the BN of urban community resilience for PHEs.

Root node ID	Failure probability	Non-failure probability
EnvS1	0.353502	0.646498
EnvS2	0.513808	0.486192
EnvS3	0.303115	0.696885
EnvS4	0.551406	0.448594
EnvR1	0.678637	0.321363
EnvR2	0.410195	0.589805
EnvR3	0.322598	0.677402
EnvR4	0.375086	0.624914
SocP1	0.295805	0.704195
SocP2	0.384659	0.615341
SocS1	0.421253	0.578747
SocS2	0.555276	0.444724
SocS3	0.337594	0.662406
SocS5	0.362025	0.637975
SocS6	0.271336	0.728664
SocR1	0.318983	0.681017
SocR2	0.57406	0.42594
SocR4	0.647044	0.352956
SocR6	0.486191	0.513809
SocR7	0.256169	0.743831
EcoS1	0.342277	0.657723
EcoS2	0.587509	0.412491
EcoS3	0.283614	0.716386
EcoS4	0.405252	0.594748
EcoR1	0.616297	0.383703
EcoR2	0.242033	0.757967
EcoR3	0.265175	0.734825
EcoR4	0.527876	0.472124

Based on the prior probabilities obtained from the fuzzy comprehensive evaluation and the Leaky Noisy-OR model, the conditional probabilities of the non-root nodes in the BN of resilience are calculated using Equation 15. Table 6 presents the conditional probability for the non-root node “Community emergency management capability,” while the conditional probability tables for the remaining non-root nodes can be found in Appendix 3.

**Table 6:** Conditional probability table of the non-root node “SocR5.”

SocR4	SocR6	SocR7	Failure probability	Non-failure probability
State 0	State 0	State 0	0.1	0.9
State 0	State 0	State 1	0.352674	0.647326
State 0	State 1	State 0	0.398835	0.601165
State 0	State 1	State 1	0.567611	0.432389
State 1	State 0	State 0	0.620758	0.379242
State 1	State 0	State 1	0.72723	0.27277
State 1	State 1	State 0	0.746681	0.253319
State 1	State 1	State 1	0.8178	0.1822

#### 3.3.3 Key resilience failure chains based on backward diagnostic inference

The BN model structure was manually constructed using Netica software and the node probabilities were imported. The resilience value of urban community resilience for PHEs was evolved through positive causality. The results showed that the probability of non-failure in resilience within Nanjing is 39.6%, with the non-failure probabilities for social, environmental, and economic resilience in communities being 37.8, 43.3, and 49.5%, respectively. The failure probabilities of the seven intermediate nodes introduced based on the PSR theory range from 50 to 60%. The failure probabilities of response resilience, with the exception of the economic system, are slightly higher than those of state resilience, with the highest being the environmental response failure probability (61.7%) and the social response failure probability (58%). This suggests that, compared to the community’s ability to defend against and respond to the pandemic using inherent resources, grassroots communities in Nanjing lack the capacity and experience to comprehensively mobilize social forces and rapidly restore normal order in environmental facilities following PHEs.

**Figure 4:**
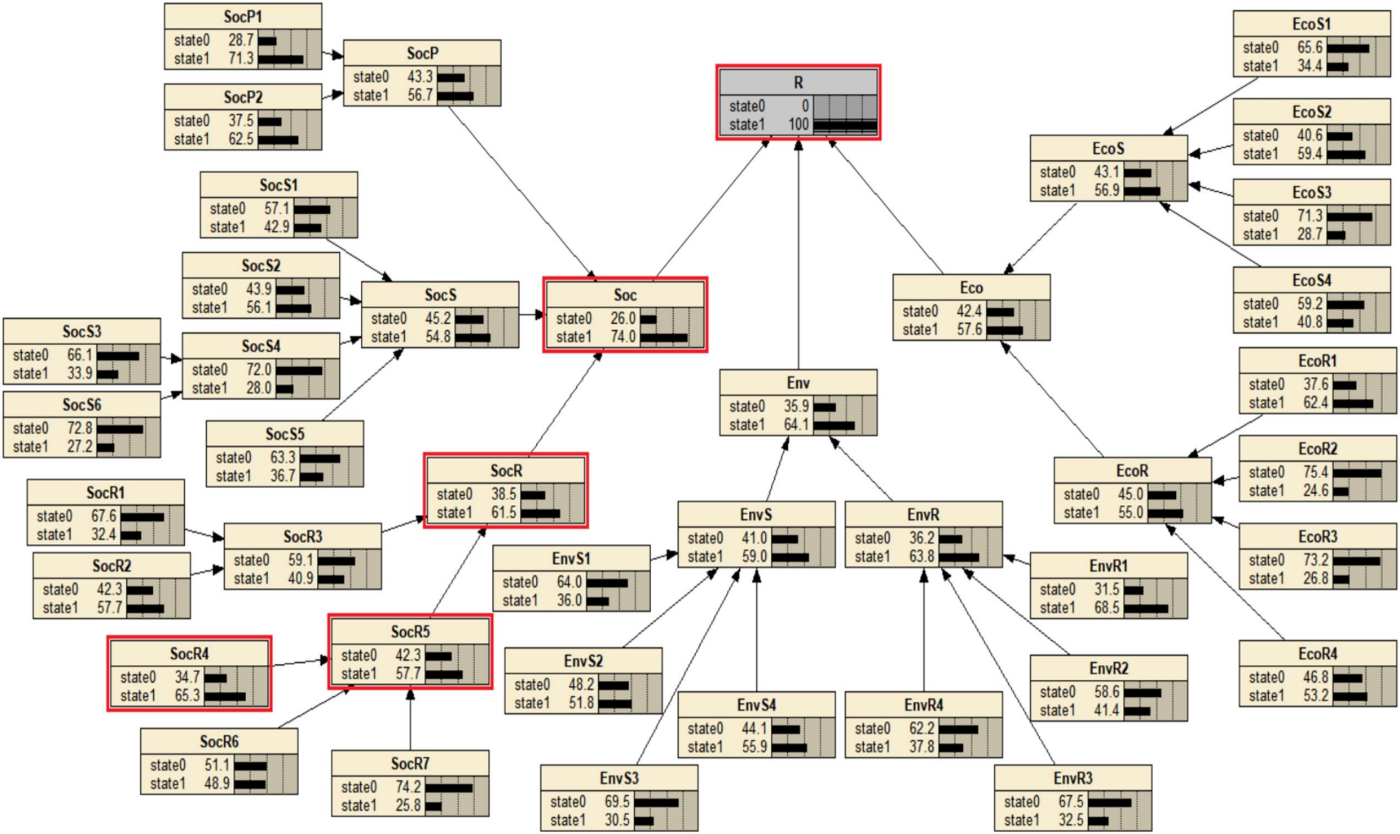
Reverse diagnosis of BN for urban community resilience for PHEs.

This study employs backward diagnostic analysis using BN to identify the most critical causal chain leading to the complete failure of urban community resilience in response to PHEs, providing a foundation for subsequent adjustments and improvements aimed at addressing weak factors. In NETICA software, the probability *P* (Resilience = state1) was set to 100%, and the backward diagnostic results for each node’s probability are presented in Figure 4. When resilience completely fails, the community’s social resilience system exhibits the highest failure probability, reaching 74%. Among the parent nodes of urban community resilience, social response has the highest failure probability. As a result, the most critical causal chain leading to resilience failure is: “Social network relationships → Community emergency management capability → Social response → Social resilience → Urban community resilience for PHEs.” This finding underscores that well-developed social networks enable individuals to efficiently share critical information and collaboratively solve problems during crises, thereby strengthening collective action capacity. Robust social network ties not only facilitate resource sharing but also enhance residents’ compliance with and implementation of containment measures, significantly improving communities’ responsiveness to PHEs (16). Moreover, communities with strong social networks demonstrate superior capabilities in mobilizing residents and social organizations, thereby establishing agile emergency management systems capable of rapid response (23). Supported by cohesive social networks and enhanced emergency management capacity, communities can implement timely interventions and develop systematic, comprehensive response mechanisms. Ultimately manifested as social resilience, these networked interactions reflect the enduring impacts of social capital and institutional preparedness on communities’ adaptive capacities. Through these relational pathways, communities maintain essential functionality post-crisis while progressively restoring stability.

At the same time, this causal chain highlights a significant issue: social networks primarily focused on entertainment and services often struggle to transform spontaneously into community emergency resources or to effectively exercise self-governance. The core issue lies in the fact that residents, as the main participants, typically engage individually or passively, seldom participating continuously and proactively in collective activities based on the community’s shared interests. As a result, it is challenging to establish cooperative, partnership-based social network relationships, which naturally impedes the community’s ability to organize and respond effectively during sudden disasters.

This diagnostic analysis serves as a crucial reminder for urban communities, like the case study, emphasizing the importance of fostering cooperation and communication among key stakeholders. Strengthening these relationships is essential for enhancing the community’s capacity to function as an effective emergency organization during crises, as well as improving its responsiveness following PHEs.

### 3.4 Optimization suggestions for urban community resilience for PHEs

This study further employs Sensitivity Analysis, Dynamic Bayesian Network (DBN) simulation, and Importance Analysis to optimize strategies for enhancing community resilience.

#### 3.4.1 Static optimization of urban community resilience based on sensitivity analysis

In this study, the sensitivity analysis model is expressed as *y* = *f* (*x*_1_, *x*_2_, …, *x*_n_), where *x_i_* represents the *i*-th root node. When the non-failure probability of *x_i_* varies within its defined range by a specified increment, the impact of each root node’s variation on the resilience output is quantitatively assessed. This impact is denoted by the sensitivity coefficient *S*. A larger value of *S* indicates a greater influence of changes in that particular factor, signifying a higher sensitivity of community resilience to it. The sensitivity coefficient *S* is computed as shown in Equation 16.


(16) $$S=\frac{R_{i}^{r}-R_{i}^{l}}{P_{i}^{r}-P_{i}^{l}}$$

Where $P_{i}^{l}$ and $P_{i}^{r}$ represent the non-failure probability values of the *i*-th root node at the left and right ends of the variation interval, respectively. $R_{i}^{l}$ and $R_{i}^{r}$ represent the non-failure probabilities of urban community resilience for PHEs, social system resilience, environmental system resilience, and economic system resilience when the non-failure probability of the *i*-th root node is $P_{i}^{l}$ and $P_{i}^{r}$ , respectively.

**Figure 5:**
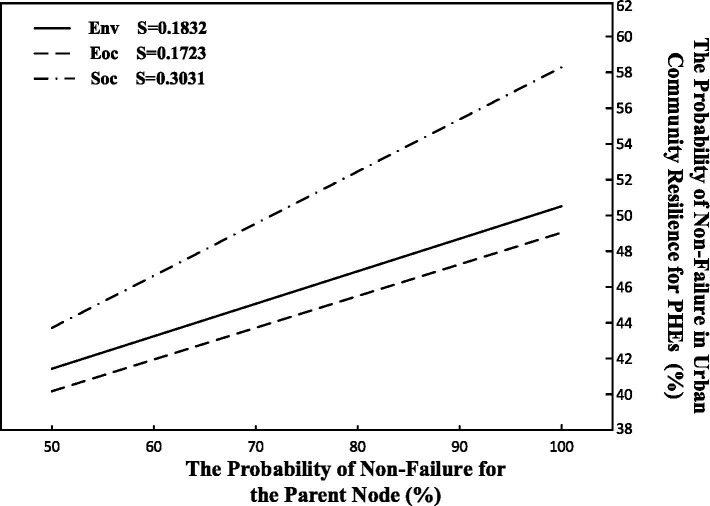
Sensitivity analysis of the parent node of urban community resilience for PHEs.

Based on the resilience measurement results from forward causal evolution, the highest non-failure probability (state 0) among the three parent nodes of resilience is 49.5%. The initial non-failure probability for the three parent nodes—community social, environmental, and economic resilience—is set at 50%, and gradually increased to 1 in increments of 10%. The changes in the non-failure probability of urban community resilience for PHEs (R) are shown in Figure 5. The slopes of the three lines represent the sensitivity coefficients of the different parent nodes, which are calculated using Equation 16. From the height, slope, and trend of the lines, it is evident that community social resilience is the most sensitive factor, exerting the greatest influence on changes in urban community resilience for PHEs. This is followed by community environmental resilience and community economic resilience, both of which exhibit similar sensitivity levels. The sensitivity of the three parent nodes is positively correlated with their respective failure probabilities.

1. Community Social Resilience

**Figure 6:**
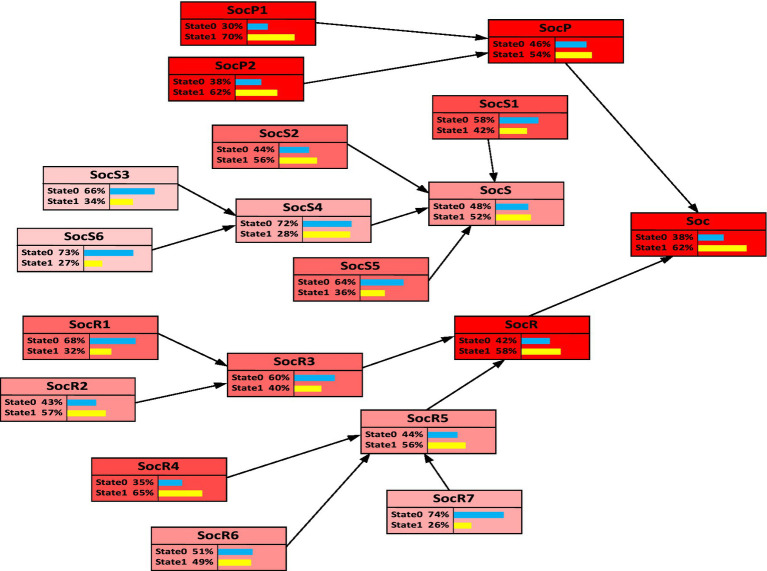
The strength level of social resilience sensitive sources.

First, we analyze the social resilience component, which exerts the most significant impact on changes in overall community resilience. In GeNIe, the “Set Target” function was utilized to determine the sensitivity levels, as shown in Figure 6.

The darker the color of the influencing factor nodes, the higher their sensitivity. Factors such as the level of PHEs, vulnerable groups, population structure, and social network relationships are depicted in darker colors, indicating greater sensitivity. Conversely, factors such as resident health status, rules and regulations, resident belongingness, and public participation appear lighter in color, reflecting lower sensitivity. Therefore, urban communities should prioritize these social factors to effectively enhance resilience to PHEs. Given that the severity of future PHEs and community population structure are objective factors that are difficult to alter through human intervention, it is essential to focus on improving regulations related to community public health and emergency management, as well as fostering greater trust and cooperation among residents.

2. Environmental Resilience and Economic Resilience

According to the forward evolution results of the BN, the highest non-failure probability (state 0) for the root nodes of environmental resilience is 69.7%, while for economic resilience, it is 75.8%. The initial non-failure probability for the root nodes of environmental resilience is set at 70%, and for economic resilience, it is set at 80%. Both are gradually increased to 1 in increments of 5%. Sensitivity coefficients were calculated for different change intervals. Factors such as sanitation state, community quality, entrance/exit management, accessibility of medical facilities, resident income, medical supplies, capital investment, and intelligent supervision have sensitivity coefficients above the average, indicating that these variables should be given particular attention and optimization recommendations.

This paper comprehensively considers both the BN reasoning probability and the magnitude of sensitivity, summarizing the static optimization strategy for urban community resilience for PHEs. The strategy is structured around the prioritization of social, environmental, and economic factors, while comparing the resilience of subsystems (state and response) under constraints of resources and time. The key strategies include: This article outlines the following optimization strategies: ① Communities should proactively develop interactive platforms, organize activities related to public health, health safety, and disaster prevention and mitigation, establish residents’ committees, encourage community participation, and promote the transformation of residents’ social networks into self-help networks. ② Communities should strengthen the protection of vulnerable groups, collaborate with community hospitals to establish green channels, and ensure timely assistance for vulnerable individuals. ③ Communities should develop management systems for pre-disaster warnings, disaster response, and post-disaster recovery. Risk assessments should be conducted through public health safety monitoring systems, and timely emergency plans and implementation strategies should be established. ④ Medical institutions should enhance medical services, provide intelligent contactless consultation equipment, collaborate with large hospitals to attract talent, and improve medical standards. ⑤ Communities should adapt the arrangement of entrances and exits, set up access control systems based on epidemic prevention levels, and regularly inspect the hygiene and safety of these areas. ⑥ Communities should establish multi-tiered material reserve mechanisms, collaborate with supermarkets, pharmacies, and other entities to ensure the supply of essential materials, and set up logistics and emergency material information-sharing platforms to strengthen material reserves and supply capabilities in emergencies. ⑦ Communities should enhance financial management, establish community funds, attract resources from residents, social enterprises, and other sources, and improve the flexibility of community funds and their ability to respond to public crises.

#### 3.4.2 DBN inference and simulation of urban community resilience for PHEs

This study introduces DBN that incorporates temporal characteristics to simulate and predict urban community resilience for PHEs, taking into account the changes in influencing factors over time. This approach enables the development of more scientifically grounded strategies for optimizing resilience from a dynamic perspective. To simplify the complex analysis of the DBN, the following reasonable assumptions are made: (1) The BN structure remains constant over time (*t*), and the process of conditional probability transitions over finite, adjacent time intervals is stable and consistent; (2) Probability transitions adhere to a first-order Markov chain, meaning that the probability distribution at time (*t* + 1) depends solely on the state at time (*t*) and is independent of any prior states. The specific modeling steps for the DBN of resilience are outlined as follows:

1. Constructing the Static Bayesian Network (*B₀*): The BN of resilience, as presented in Section 3.3 of this study, serves as *B₀*. It represents the joint probability distribution at the initial state of the DBN.
2. Constructing the Bayesian Transition Network (*B*→): A DBN combines both the BN structure and Markov assumptions to model temporal data. It decomposes the data into a series of time slices, where the node variables within each time slice form a static BN, and standard arcs represent relationships within the same time slice. Temporal arcs, on the other hand, represent relationships between nodes in different time slices and are defined by transition probabilities that describe the relationships between root nodes in adjacent time slices.
3. Determine the State Transition Probability Matrix: Define the corresponding probability transition matrices according to the different types of variables. The causes of safety risks are typically categorized into human, equipment, environmental, and management factors. In this study, the failure transition matrices for root node variables are constructed from three perspectives—human factors, physical factors, and management factors—based on the community resilience subsystems and influencing factor systems. Root nodes that lack significant temporal characteristics (e.g., the level of PHEs) are excluded from the scope of the DBN analysis. The classification of the three types of root nodes and their corresponding transition probabilities are outlined as follows:

1. Human Factor Root Nodes

Human factors refer to the behaviors, work, characteristics, emotions, and interactions of various personnel involved in community epidemic prevention and control, including community residents, community workers, and grassroots managers. It is assumed that human factors (nodes) will be in one of two states in the future: positive (0) or negative (1). Negative or non-compliant behaviors that result in errors are generally independent of random events. Assuming that the average frequency of human errors per unit time is *λ_1_*, the human factor transition probabilities, as shown in Table 7, follow a Poisson distribution (59). The root nodes for human factors include sanitation state, resident health status, resident belongingness, public service, and social network relationships.

2. Physical Factor Root Nodes

**Table 7:** State transition probability matrix of DBN.

Root node variable type	Time *t*	Time *t* + ∆*t*	
		State 0	State 1
Human node	State 0	$1-\lambda_{1}e^{-\lambda_{1}}$	$\lambda_{1}e^{-\lambda_{1}}$
	State 1	$e^{-\lambda_{1}}$	$1-e^{-\lambda_{1}}$
Physical node	State 0	$e^{-\lambda_{2}\mathrm{\Delta t}}$	$1-e^{-\lambda_{2}\mathrm{\Delta t}}$
	State 1	$1-e^{-\mathrm{\mu \Delta t}}$	$e^{-\mathrm{\mu \Delta t}}$
Management node	State 0	1	0
	State 1	*c*	1 − *c*

Physical factors refer to common community resources, infrastructure, or disaster prevention and mitigation facilities. It is assumed that physical factors (nodes) are in one of two states: normal (0) or failed (1). Community environmental resources, infrastructure, and disaster prevention facilities inevitably deteriorate over time, requiring community staff to perform regular maintenance and updates to ensure proper functioning. Assuming the facility failure rate is *λ₂* and the facility maintenance and repair rate is *μ*, the transition probabilities, as shown in Table 7, follow an exponential distribution (58). The root nodes for physical factors include accessibility of medical facilities, living support facilities, transportation robustness, public space, entrance/exit management, communication system, and intelligent supervision.

3. Management Factor Root Nodes

It is assumed that all management factors (nodes) are in one of two states: reasonable (0) or unreasonable (1). As the duration of community public health governance increases, members accumulate relevant management experience. The introduction of the enhancement coefficient *c*, as shown in Table 7, reflects the improvement in comprehensive management capabilities, such as decision-making, execution, and learning, brought about by experience accumulation. The management factor root nodes include medical supplies, capital investment, emergency shelter, rules and regulations, publicity and education, past experience, and government leadership functions.

To construct the DBN, it is necessary to set the facility failure rate *λ_2_*, the maintenance and repair rate *μ*, the frequency of human errors *λ_1_*, and the management enhancement coefficient *c*. Drawing on relevant literature and the principles of control variables, this study created 12 simulation scenarios, as shown in Table 8. Based on simulation experiments and expert insights, the parameter values of the transition probability matrices for the three root nodes were appropriately set to ensure that the dynamic simulation results of urban community resilience for PHEs are scientifically robust and accurate. In this context, *λ*₁ = 1, 4, 12 represent the probabilities of human-caused negligence occurring once a year, once a quarter, and once a month, respectively. These values cover scenarios ranging from “low-frequency and occasional” to “high-frequency and habitual.” *λ*₂ = 1/365, 12/365, 48/365 represent the probabilities of facility failure occurring once a year, once a month, and once a week, respectively. These values reflect scenarios ranging from “low-fault in new communities” to “high-fault in older communities.” *μ* = 1, 0.1, 0.01 represent the maintenance cycles of 1 day, 10 days, and 100 days, respectively, covering scenarios from “high-frequency maintenance” to “low-frequency maintenance.” Lastly, c = 1, 0.1, 0.01 represent varying levels of management intervention, ranging from “strong management intervention” to “no management intervention.”

**Table 8:** Design simulation scenarios for DBN.

Observation group	Scenario no.	*λ*_1_	*λ*_2_	*μ*	*c*
Human errors *λ*_1_	(1)	1	12/365	0.1	0.1
	(2)	4			
	(3)	12			
Facility failure rate *λ*_2_	(4)	4	1/365	0.1	0.1
	(5)		12/365		
	(6)		48/365		
Maintenance and repair rate *μ*	(7)	4	12/365	1	0.1
	(8)			0.1	
	(9)			0.01	
Management enhancement coefficient *c*	(10)	4	12/365	0.1	1
	(11)				0.1
	(12)				0.01

**Figure 7:**
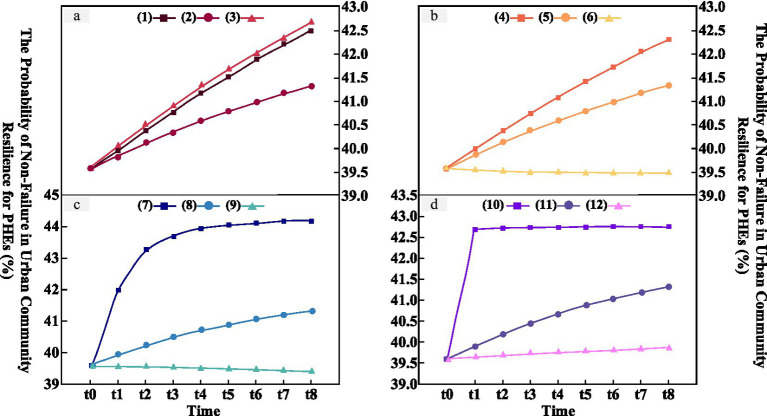
Dynamic inference simulation of urban community resilience for PHEs.

Based on the designed simulation scenarios, the original BN was transformed 8 times using GeNIe to construct a DBN for simulating and predicting resilience. The changes in resilience probability obtained from the simulation experiments across the four groups of control variables are shown in Figure 7. Group (a): Only the number of human errors (*λ_1_*) was observed. Comparing scenarios (1) and (3), scenario (2) exhibits a relatively slow upward trend in resilience, indicating that extreme situations where human errors occur either too infrequently or too frequently tend to stimulate and enhance community resilience. Conversely, occasional errors can lead to community complacency. Therefore, setting the probability of human-induced errors to once per quarter aligns more closely with practical circumstances. Group (b): Only the failure rate of facilities (*λ_2_*) was observed. Resilience in scenario (6) initially declines slowly and then stabilizes around *t_7_*, after which it begins to recover gradually. The state of resilience in this scenario is suboptimal. The lower the *λ_2_* in scenarios (4) and (5), the faster the resilience increases. Group (c): Only the maintenance and repair rate of facilities (*μ*) was observed. The higher the repair rate (*μ*), the faster the resilience grows. However, in specific cases, such as scenario (9), where facility maintenance cannot keep up with the failure rate, resilience declines at a steady rate. Group (d): Only the management enhancement coefficient (*c*) was observed. In scenario (10), when the management enhancement coefficient reaches its maximum value of 1, resilience rises sharply at *t_1_* and then remains stable over the long term, representing a rare scenario.

Based on the analysis of the simulation scenario results, expert experience, and literature references (36), it is relatively reasonable to set *λ_1_* = 4, *λ_2_* = 12/365, *μ* = 0.1, and *c* = 0. Using these parameters, the DBN for resilience was constructed. This DBN reflects the changes in the state of each influencing factor over time, as well as the resilience level at different time points. It enables dynamic simulation and prediction of urban community resilience for PHEs, providing a basis for formulating long-term dynamic strategies to enhance urban community resilience.

#### 3.4.3 Dynamic optimization of urban community resilience based on importance analysis

Bayesian network importance analysis is a method used to assess the influence of initial nodes on outcome nodes. This technique determines the significance of variables by considering both the structure and parameters of the BN, revealing the degree to which initial variables impact the target outcomes. Importance analysis is commonly employed to identify key factors, optimize model design, and enhance efficiency. Its main advantage lies in accounting for dependencies between variables, thus preventing the neglect of crucial interaction effects. Additionally, it can handle various uncertainties, such as model structure uncertainty and data variability, and is applicable to complex nonlinear models without assuming specific model forms or relying on similar methods. Bayesian network importance analysis is particularly useful for evaluating how uncertain factors affect model outcomes in fields such as risk analysis, decision support, and knowledge discovery (37, 38).

This study evaluates the significance of each root node factor in urban community resilience for PHEs. Using BN inference algorithms, three types of importance parameters—probabilistic, critical, and structural—are derived for the root nodes. Subsequently, by integrating DBN analysis, the temporal variations in the importance of influencing factors are assessed. Based on these results, strategies are proposed to facilitate the dynamic enhancement of urban community resilience. The specific steps are outlined as follows:

1. Calculation of Probabilistic Importance

Probabilistic importance (PI) refers to the degree to which a unit change in the failure probability of a specific root node influences the failure probability of the leaf node. It is denoted as PI and calculated using Equation 17. PI effectively categorizes the importance of factors from a sensitivity perspective. By calculating the PI for each root node, the factors that most effectively and rapidly reduce the failure rate of urban community resilience can be identified.


(17) $${\mathrm{PI}}_{i}=P\;(R=1|X_{i}=1)-P\;(R=1|X_{i}=0)\;$$

2. Calculation of Critical Importance

Critical importance (CI) is defined as the ratio of the change rate in the failure probability of the leaf node to the change rate in the failure probability of a specific root node. It is denoted as CI and calculated using Equation 18. Compared to PI, CI provides a more comprehensive metric by evaluating the significance of factors from both sensitivity and failure rate perspectives. A higher CI indicates that the root node (influencing factor) is more likely to trigger the failure of the leaf node (resilience). Additionally, it suggests that optimizing this influencing factor may be more feasible, as it is generally easier to reduce the failure probability of a root node with a high initial failure probability than one with a low initial failure probability.


(18) $${\mathrm{CI}}_{i}=\frac{P(X_{i}=1)\times {\mathrm{PI}}_{i}}{P(R=1)}$$

3. Calculation of Structural Importance

Structural importance (SI) is defined as the impact of the failure of a particular root node on the probability of community resilience failure, assuming that the failure probabilities of all other root nodes are held constant. SI is calculated using Equation 19 and reflects the significance of each influencing factor node within the BN structure. This metric provides valuable insight for enhancing the structure of the BN model for urban community resilience in the context of PHEs.


(19) $${\mathrm{SI}}_{i}=P\;(\begin{array}{l} R=1|,X_{i}=1|,\\ P\;(X_{j}=1)=0.5 \end{array})-P\;(\begin{array}{l} R=1|,X_{i}=0|,\\ P\;(X_{j}=1)=0.5 \end{array})\;$$

In the above three equations, *x_i_* represents the *i*-th root node; R represents the leaf node (urban community resilience for PHEs); *X_j_* denotes any root node other than *x_i_*; 1 indicates node failure; and 0 indicates node non-failure.

The corresponding probability values were obtained by adjusting the BN model in GeNIe software and calculating them according to Equations 17–19, as shown in Appendix 4. The results indicate that factors with high PI also tend to have high CI (e.g., medical supplies, entrance/exit management, accessibility of medical facilities), whereas factors with low CI generally exhibit low PI (e.g., resident educational level, publicity and education, public space). Notably, there is no significant difference in their overall rankings.

The top 10 SI rankings are predominantly occupied by factors from the social and environmental dimensions, indicating that the economic dimension is relatively less significant. When simplifying the BN model structure, factors with lower SI should be prioritized for consideration. Based on the formula and the concept of structural importance, SI is solely determined by the constructed BN structure. Since the DBN structure in this study remains unchanged, this indicator is not influenced by time.

**Figure 8:**
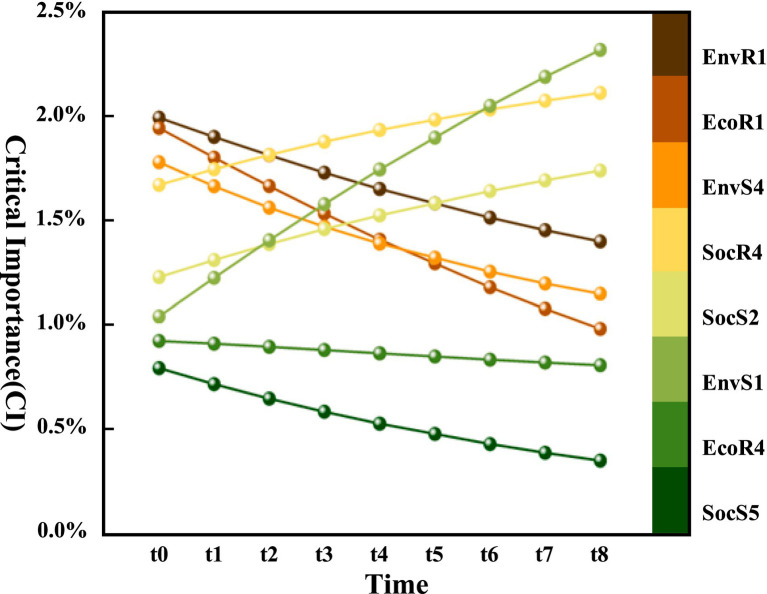
Dynamic changes in CI of some influencing factors.

Among the three indicators—PI, CI, and SI—CI provides a more comprehensive reflection of issues and is therefore relatively more significant. Consequently, this study prioritizes the analysis of the temporal changes in this indicator. Based on the initial CI ranking at time t0, the top eight influencing factors with temporal characteristics were selected. The CI trends over the eight transitions of the DBN are shown in Figure 8. The critical importance of sanitation state, resident health status, and social network relationships increases monotonically over time, with sanitation state showing the fastest and most significant growth. Conversely, the critical importance of accessibility of medical facilities, medical supplies, entrance/exit management, and rules and regulations decreases monotonically, while the trend of intelligent supervision remains relatively stable.

**Figure 9:**
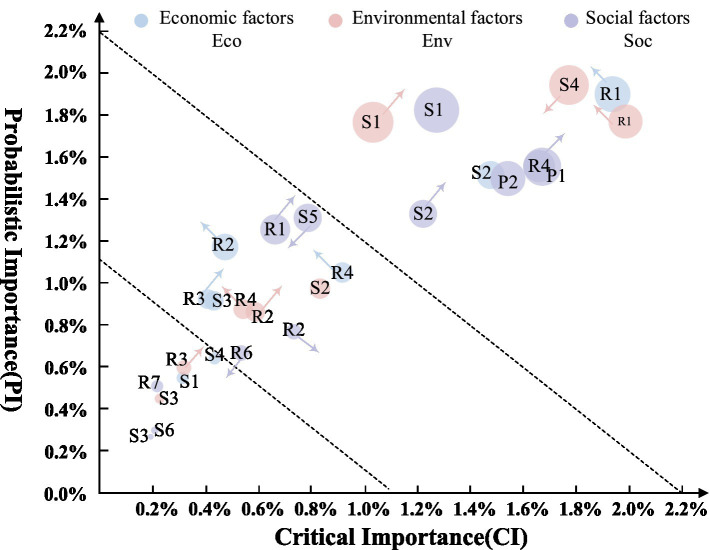
Importance analysis results of community epidemic resilience factors.

In summary, the comparison results of the three importance parameters of root nodes in the BN of resilience at time *t*_0_ are shown in Figure 9. The larger the volume of the bubble, the higher the SI. The direction of the arrows indicates the dynamic changes in PI and CI of the influencing factors over time. The bubbles are divided into three levels by dashed lines, with importance decreasing from the outer to the inner levels. In the long-term process of building resilient communities to adapt to and prevent PHEs, the influencing factors located at the top right of the bubble chart, especially those with arrows pointing upwards to the top right, should be prioritized for optimization. These include factors such as sanitation state, social network relationships, and resident health status. In the medium term, attention should be given to enhancing the factors between the two dashed lines, which also have arrows pointing upwards to the top right, such as the communication system and emergency shelter. Finally, in the short term, consider the influencing factors near the inner dashed line with a trend of crossing levels, such as past experience and living supporting facilities.

Based on the results of the importance analysis, the focus of community work should be adjusted in response to the changing importance of influencing factors over different time periods during long-term public health crisis prevention and control. This paper proposes a dynamic optimization strategy for enhancing urban community resilience for PHEs, from the perspectives of the short, medium, and long term. ① The initial strategy prioritizes “social network development + environmental health management.” Governments incentivize businesses to engage in community governance during PHEs, ensuring the continuous flow and supply of resources during outbreaks. Neighborhood committee activities cultivate close-knit, mutually supportive relationships among residents, while communities enhance sanitation management by promptly clearing waste and debris. ② Mid-term strategies emphasize “intelligent platform integration + spatial function adaptation,” combining community management with medical and daily services to create seamless living ecosystems. The flexible repurposing of spaces such as parking lots and hotels allows for their conversion into emergency medical facilities and material storage areas. ③ Long-term strategies focus on the “simultaneous enhancement of both software and hardware.” Managers improve governance efficiency through skill development and resident feedback, while upgrading essential living infrastructure. This fosters a living services network that balances equity and pandemic preparedness. By dynamically adjusting priorities, communities establish a resilience-building pathway that spans the entire “prevention-response-recovery” cycle.

## 4 Conclusion

This study follows the research paradigm of “comprehensive multi-dimensional identification—bidirectional inference measurement—effective targeted optimization,” and has drawn the following conclusions:

The study developed a resilience model for urban community resilience for PHEs based on the PSR-SENCE framework, identifying 31 influencing factors. The DEMATEL method was then employed to identify 10 key causal pathways in social resilience, providing a scientific foundation for comprehensive resilience assessment. Using BN, the study conducted a case analysis of the response process of a disaster-resistant model community in Nanjing to the epidemic. The results revealed that the most critical failure chain in terms of resilience was: “Social network relationships → Community emergency management capability → Social response → Social resilience → urban community resilience for PHEs.” Under PHEs, urban community resilience for PHEs is significantly influenced by social network relationships. Individuals share information and collaborate through social networks, while communities can more effectively mobilize residents and leverage emergency management capabilities via these networks to respond promptly and implement measures.

This study employed scenario simulation and importance analysis, concluding that the social dimension is central to resilience building and plays a pivotal role in establishing effective emergency response systems. To promote continuous enhancement of urban community resilience for PHEs, the study proposes short-term, medium-term, and long-term dynamic optimization strategies for urban community resilience, enabling adaptation to evolving PHEs and changing internal community conditions.

In comparison to existing studies, this research comprehensively considers the combined attributes of resilience in systems and processes, and constructs a city community resilience model under sudden PHEs. Additionally, this study introduces DBN for simulation, offering new perspectives and tools for the long-term dynamic management and optimization of community resilience. This work provides valuable guidance for urban communities in formulating epidemic prevention and disaster mitigation plans, thereby enhancing their resilience and improving their ability to manage future uncertainties and risks.

This study has several limitations: ① The limited sample size and the subjectivity of expert opinions hinder the ability of this study to fully represent all communities. Significant variations, particularly across different regions or types of communities, may affect the generalizability and representativeness of the findings. Future research should expand the sample size and scope to include a broader range of regions, scales, and community types, and incorporate digital technologies such as blockchain and big data to enhance the robustness of the conclusions. ② The Bayesian network model relies on several assumptions, which may not fully capture the complexity of real-world conditions. Future studies could introduce additional models for validation.

## Data Availability

The original contributions presented in the study are included in the article/Supplementary material, further inquiries can be directed to the corresponding author.
