# Supplementary material for: Measuring and optimizing the urban community resilience against public health emergencies: a case study in Nanjing, China
Source: Front Public Health. 2025 Oct 16;13:1691666. doi: 10.3389/fpubh.2025.1691666 (PMC12571826; doi:10.3389/fpubh.2025.1691666)
Supplement: Supplementary file 1 [file Data_Sheet_1.DOCX]

Appendix 1

Research questionnaire on the relationship of factors influencing

Urban Community Resilience for PHEs

Dear Experts:

Thanks for being able to participate in this questionnaire on your busy schedule. This research group is conducting the research on Urban Community Resilience for PHEs, resilience refers to the ability of a community to withstand or resist the impact of a disaster event, and to maintain or rapidly restore community functions, strengthening urban community construction with the concept of resilience as the core, provides new ideas to enhance the community prevention and control capacity of PHEs.

The purpose of this questionnaire is: to analyse the role relationship between the influencing factors of Urban Community Resilience for PHEs. Please score it according to the 0-4 scoring method, where the scale ranges from “no influence” to “low,” “medium,” “high,” and “very high” influence, and the greater the influence of the indicator in the row on the left side of the table on the indicator in the column above, the higher the score. The research team ensures that the information in this questionnaire is used for academic research only, and we recommend that you use a computer or tablet to fill it out. Thank you again for your participation!

Relationship Scoring Scale for Factors Influencing Social Resilience in Urban Community Resilience for PHEs

| **Influencing factors** | P1 | P2 | S1 | S2 | S3 | S4 | S5 | S6 | R1 | R2 | R3 | R4 | R5 | R6 | R7 |
| --- | --- | --- | --- | --- | --- | --- | --- | --- | --- | --- | --- | --- | --- | --- | --- |
| Level of PHEs (P1) | — |  |  |  |  |  |  |  |  |  |  |  |  |  |  |
| Vulnerable groups (P2) |  | — |  |  |  |  |  |  |  |  |  |  |  |  |  |
| Population structure  (S1) |  |  | — |  |  |  |  |  |  |  |  |  |  |  |  |
| Resident health status  (S2) |  |  |  | — |  |  |  |  |  |  |  |  |  |  |  |
| Resident educational level (S3) |  |  |  |  | — |  |  |  |  |  |  |  |  |  |  |
| Risk awareness (S4) |  |  |  |  |  | — |  |  |  |  |  |  |  |  |  |
| Rules and regulations  (S5) |  |  |  |  |  |  | — |  |  |  |  |  |  |  |  |
| Publicity and education  (S6) |  |  |  |  |  |  |  | — |  |  |  |  |  |  |  |
| Resident belongingness  (R1) |  |  |  |  |  |  |  |  | — |  |  |  |  |  |  |
| Public service (R2) |  |  |  |  |  |  |  |  |  | — |  |  |  |  |  |
| Public participation  (R3) |  |  |  |  |  |  |  |  |  |  | — |  |  |  |  |
| Social network relationships (R4) |  |  |  |  |  |  |  |  |  |  |  | — |  |  |  |
| Community emergency management capability  (R5) |  |  |  |  |  |  |  |  |  |  |  |  | — |  |  |
| Past experience (R6) |  |  |  |  |  |  |  |  |  |  |  |  |  | — |  |
| Government leadership functions (R7) |  |  |  |  |  |  |  |  |  |  |  |  |  |  | — |

Appendix 2

Research questionnaire on the relationship of factors influencing

Urban Community Resilience for PHEs

Dear Experts:

Thanks for being able to participate in this questionnaire on your busy schedule. This research group is conducting the research on Urban Community Resilience for PHEs, resilience refers to the ability of a community to withstand or resist the impact of a disaster event, and to maintain or rapidly restore community functions, strengthening urban community construction with the concept of resilience as the core, provides new ideas to enhance the community prevention and control capacity of PHEs.

The purpose of this questionnaire is to analyse and judge the performance of the various components of the community system, and to provide a reference for measuring the Urban Community Resilience for PHEs. This questionnaire consists of single-choice questions, and we hope that you will choose the appropriate option according to the actual situation and work experience of your community. The research team guarantees that the information in this questionnaire will only be used for academic research and will not be disclosed. Your answers are important to us, and we recommend that you use a computer or tablet to complete the questionnaire. Thank you again for your participation!

**Part I - Personal Information**

1. Your gender:

| ○Male | ○Female |
| --- | --- |

2. Your education level:

| ○College degree or below | ○Bachelor’s degree | ○Master’s degree | ○Doctor degree or above |
| --- | --- | --- | --- |

3. How long have you worked or researched in areas such as “urban communities” or “Public health emergency management” or “disaster resilience”:

| ○Within 1 year | ○1-3 years | ○3-5 years | ○more than 5 years |
| --- | --- | --- | --- |

4. Your work department or position:

| ○Comprehensive Management | ○Party building | ○People’s livelihood |
| --- | --- | --- |
| ○Health care | ○Higher education | ○Other |

5. What is your understanding of urban community resilience or emergency response (policies, technologies, knowledge, etc.):

| ○Hardly understand | ○Understand a little | ○Generally understand |
| --- | --- | --- |
| ○Better understand | ○Very understand |  |

**Part II - Factor Link Performance**

**Basic concept:**

(1) Urban Community Resilience for PHEs: the community’s prevention of PHEs such as infectious diseases, and the use of internal and external resources to resist negative impacts, maintain and restore community functions, protect public health security, and promote self-adaptation and self-improvement. Urban Community Resilience is composed of three subsystems: environment, economy and society.

(2) Environment refers to the community natural conditions, building space, physical facilities, etc.

(3) Economy refers to the flow of resources and information for the survival and development of communities, the coordination of epidemic prevention policies and the improvement of public health.

(4) Society refers to the characteristics of community population, humanistic spirit, man-governed management and so on.

(5) The resilience subsystem corresponding to the pre-disaster, mid-disaster and post-disaster stages can be divided into three dimensions: stress, state and response:

① Stress resilience refers to the ability of communities to withstand disaster-causing factors and disaster-bearing environments in the face of unknown PHEs.

② State resilience refers to the disaster bearing level of community socio-economic-environmental systems in PHEs, which is embodied by the robustness and redundancy of inherent resources.

③ Response resilience refers to the ability of social, economic and environmental systems to take measures and learn to recover under the impact of PHEs, which is embodied in the characteristics of strategy and timeliness.

**Guidance:**

Based on work experience and the actual situation of the community, judge the “possibility” of two questions:

(1) The possibility of “a factor failure”.

(2) The possibility of “failure of the pointed factor when only one factor fails, and other factors do not fail”.

**【Section 1 Environmental resilience】**

Environmental state factors performance:

EnvS1 fails: The community garbage disposal, cleaning and disinfection are not in place, and the environmental sanitation is dirty and messy, which increases the risk of disease transmission.

EnvS2 fails: The plot ratio, greening rate and sunshine ventilation level of the community do not meet the standard design.

EnvS3 fails: The per capita public space area of the community is very small or there are few green spaces, squares and parks in and around the community.

EnvS4 fails: The community entrance and exit planning is unreasonable, the closed management is difficult, and the epidemic prevention function cannot be well played.

EnvS fails: The community does not have a healthy, safe and comfortable environment to withstand the disturbance of PHEs.

| Environmental state factors performance | Probability | | | | | | |
| --- | --- | --- | --- | --- | --- | --- | --- |
|  | Very low | A little low | Low | Medium | High | A little High | Very High |
| EnvS1 fails |  |  |  |  |  |  |  |
| EnvS2 fails |  |  |  |  |  |  |  |
| EnvS3 fails |  |  |  |  |  |  |  |
| EnvS4 fails |  |  |  |  |  |  |  |
| When only EnvS1 fails,  EnvS fails |  |  |  |  |  |  |  |
| When only EnvS2 fails,  EnvS fails |  |  |  |  |  |  |  |
| When only EnvS3 fails,  EnvS fails |  |  |  |  |  |  |  |
| When only EnvS4 fails,  EnvS fails |  |  |  |  |  |  |  |

Environmental response factors performance:

EnvR1 fails: The number and scale of medical facilities around the community cannot meet the needs of residents for medical treatment in case of epidemic.

EnvR2 fails: Redundant venues, hotels and multi-function centers around the community cannot be converted into emergency facilities.

EnvR3 fails: supermarkets, pharmacies, express stations, sports facilities and community service stations around the community cannot serve properly.

EnvR4 fails: The community road is not usual, tidy, there is no emergency passage, and the traffic around the community is not convenient.

EnvR fails: The community fails to respond to the needs of the war and restore normalcy quickly due to the lack of complete and accessible environmental facilities.

| Environmental response factors performance | Probability | | | | | | |
| --- | --- | --- | --- | --- | --- | --- | --- |
|  | Very low | A little low | Low | Medium | High | A little High | Very High |
| EnvR1 fails |  |  |  |  |  |  |  |
| EnvR2 fails |  |  |  |  |  |  |  |
| EnvR3 fails |  |  |  |  |  |  |  |
| EnvR4 fails |  |  |  |  |  |  |  |
| When only EnvR1 fails,  EnvR fails |  |  |  |  |  |  |  |
| When only EnvR2 fails,  EnvR fails |  |  |  |  |  |  |  |
| When only EnvR3 fails,  EnvR fails |  |  |  |  |  |  |  |
| When only EnvR4 fails,  EnvR fails |  |  |  |  |  |  |  |

Environmental resilience factors performance:

EnvS fails: The community does not have a healthy, safe and comfortable environment to withstand the disturbance of PHEs.

EnvR fails: The community fails to respond to the needs of the war and restore normalcy quickly due to the lack of complete and accessible environmental facilities.

Env fails: It is difficult for urban communities to use environmental resources to prevent, absorb, mitigate the impact of PHEs and protect public health security.

| Environmental resilience factors performance | Probability | | | | | | |
| --- | --- | --- | --- | --- | --- | --- | --- |
|  | Very low | A little low | Low | Medium | High | A little High | Very High |
| When only EnvS fails,  Env fails |  |  |  |  |  |  |  |
| When only EnvR fails,  Env fails |  |  |  |  |  |  |  |

**【Section 2 Economic resilience】**

Economic state factor performance:

EcoS1 fails: The employment rate of the community residents is low, and the occupation is very unstable.

EcoS2 fails: The per capita disposable monthly income of a community resident falls below the city or urban average.

EcoS3 fails: Fewer residents have social security or other insurance.

EcoS4 fails: Fewer residents with high-value fixed assets such as cars and houses, low rents for neighborhood shops and fewer commercial buildings.

EcoS fails: Communities lack the stable and diverse economic capacity to withstand PHEs.

| Economic state  factor performance | Probability | | | | | | |
| --- | --- | --- | --- | --- | --- | --- | --- |
|  | Very low | A little low | Low | Medium | High | A little High | Very High |
| EcoS1 fails |  |  |  |  |  |  |  |
| EcoS2 fails |  |  |  |  |  |  |  |
| EcoS3 fails |  |  |  |  |  |  |  |
| EcoS4 fails |  |  |  |  |  |  |  |
| When only EcoS1 fails,  EcoS fails |  |  |  |  |  |  |  |
| When only EcoS2 fails,  EcoS fails |  |  |  |  |  |  |  |
| When only EcoS3 fails,  EcoS fails |  |  |  |  |  |  |  |
| When only EcoS4 fails,  EcoS fails |  |  |  |  |  |  |  |

Economic response factor performance:

EcoR1 fails: Communities are short of supplies during the pandemic.

EcoR2 fails: The community does not have sufficient funds for epidemic prevention or special disaster prevention and mitigation funds.

EcoR3 fails: The community lacks online communication platforms and information exchange is not smooth.

EcoR4 fails: The community is not equipped with intelligent epidemic prevention facilities, which cannot timely monitor and warn the PHEs.

EcoR fails: The community fails to properly allocate economic goods and use technology, and fails to respond to the needs of the war and restore normalcy quickly.

| Economic response factor performance | Probability | | | | | | |
| --- | --- | --- | --- | --- | --- | --- | --- |
|  | Very low | A little low | Low | Medium | High | A little High | Very High |
| EcoR1 fails |  |  |  |  |  |  |  |
| EcoR2 fails |  |  |  |  |  |  |  |
| EcoR3 fails |  |  |  |  |  |  |  |
| EcoR4 fails |  |  |  |  |  |  |  |
| When only EcoR1 fails,  EcoR fails |  |  |  |  |  |  |  |
| When only EcoR2 fails,  EcoR fails |  |  |  |  |  |  |  |
| When only EcoR3 fails,  EcoR fails |  |  |  |  |  |  |  |
| When only EcoR4 fails,  EcoR fails |  |  |  |  |  |  |  |

Economic resilience performance:

EcoS fails: Communities lack the stable and diverse economic capacity to withstand PHEs.

EcoR fails: The community fails to properly allocate economic goods and use technology, and fails to respond to the needs of the war and restore normalcy quickly.

Eco fails: It is difficult for urban communities to use economic resources to prevent, absorb and mitigate the impact of PHEs and protect public health security.

| Economic resilience performance | Probability | | | | | | |
| --- | --- | --- | --- | --- | --- | --- | --- |
|  | Very low | A little low | Low | Medium | High | A little High | Very High |
| When only EcoS fails,  Eco fails |  |  |  |  |  |  |  |
| When only EcoR fails,  Eco fails |  |  |  |  |  |  |  |

**【Section 3 Social resilience】**

Public participation performance:

SocR1 fails: Members have a weak sense of trust and belongingness to the community, and fail to form community consensus or community culture.

SocR2 fails: The community fails to provide proper public services, or the residents are not satisfied with their public services.

SocR3 fails: Residents have a low willingness to participate in and cooperate with community epidemic prevention, and the community hardly gets help from social organizations.

| Public participation performance | Probability | | | | | | |
| --- | --- | --- | --- | --- | --- | --- | --- |
|  | Very low | A little low | Low | Medium | High | A little High | Very High |
| SocR1 fails |  |  |  |  |  |  |  |
| SocR2 fails |  |  |  |  |  |  |  |
| When only SocR1 fails,  SocR3 fails |  |  |  |  |  |  |  |
| When only SocR2 fails,  SocR3 fails |  |  |  |  |  |  |  |

Community emergency management capability performance:

SocR4 fails: The frequency of contact and mutual assistance between community members, the community and the outside world is low.

SocR6 fails: The community failed to learn from, absorb experience, and implement special improvement projects for the epidemic.

SocR7 fails: The work guidance, management deployment, and personage support of the government or grassroots authorities for community epidemic prevention are weak.

SocR5 fails: The community fails to set up epidemic prevention emergency team in time, or the division of emergency team is chaotic.

| Community emergency management capability performance | Probability | | | | | | |
| --- | --- | --- | --- | --- | --- | --- | --- |
|  | Very low | A little low | Low | Medium | High | A little High | Very High |
| SocR4 fails |  |  |  |  |  |  |  |
| SocR6 fails |  |  |  |  |  |  |  |
| SocR7 fails |  |  |  |  |  |  |  |
| When only SocR4 fails,  SocR5 fails |  |  |  |  |  |  |  |
| When only SocR6 fails,  SocR5 fails |  |  |  |  |  |  |  |
| When only SocR7 fails,  SocR5 fails |  |  |  |  |  |  |  |

Social response factor performance:

SocR3 fails: Residents have a low willingness to participate in and cooperate with community epidemic prevention, and the community hardly gets help from social organizations.

SocR5 fails: The community fails to set up epidemic prevention emergency team in time, or the division of emergency team is chaotic.

SocR fails: The ability and willingness of the community and social parties to act is insufficient, which fails to respond to the needs in a timely manner and restore normalcy quickly.

| Social response  factor performance | Probability | | | | | | |
| --- | --- | --- | --- | --- | --- | --- | --- |
|  | Very low | A little low | Low | Medium | High | A little High | Very High |
| When only SocR3 fails,  SocR fails |  |  |  |  |  |  |  |
| When only SocR5 fails,  SocR fails |  |  |  |  |  |  |  |

Risk awareness performance:

SocS3 fails: The educational level and cultural accomplishment of residents are generally low.

SocS6 fails: There are very few public health related advocacy, training and information activities in the community.

SocS4 fails: Residents do not know the common sense of PHEs and a low level of preparedness.

| Risk awareness performance | Probability | | | | | | |
| --- | --- | --- | --- | --- | --- | --- | --- |
|  | Very low | A little low | Low | Medium | High | A little High | Very High |
| SocS3 fails |  |  |  |  |  |  |  |
| SocS6 fails |  |  |  |  |  |  |  |
| When only SocS3 fails,  SocS4 fails |  |  |  |  |  |  |  |
| When only SocS6 fails,  SocS4 fails |  |  |  |  |  |  |  |

(Few remaining questionnaire contents are omitted)

Appendix 3

Urban Community Resilience for PHEs BN node conditional probability table

Table 1. Conditional probability table of the node “SocS4”

| SocS3 | SocS6 | Failure Probability | Non-failure Probability |
| --- | --- | --- | --- |
| State 0 | State 0 | 0.1 | 0.9 |
| State 0 | State 1 | 0.318983 | 0.681017 |
| State 1 | State 0 | 0.470052 | 0.529948 |
| State 1 | State 1 | 0.598996 | 0.401004 |

Table 2. Conditional probability table of the node “SocR3”

| SocR1 | SocR2 | Failure Probability | Non-failure Probability |
| --- | --- | --- | --- |
| State 0 | State 0 | 0.1 | 0.9 |
| State 0 | State 1 | 0.394626 | 0.605374 |
| State 1 | State 0 | 0.59026 | 0.40974 |
| State 1 | State 1 | 0.724393 | 0.275607 |

Table 3. Conditional probability table of the node “SocP”

| SocP1 | SocP2 | Failure Probability | Non-failure Probability |
| --- | --- | --- | --- |
| State 0 | State 0 | 0.893994 | 0.106006 |
| State 0 | State 1 | 0.712544 | 0.287456 |
| State 1 | State 0 | 0.668105 | 0.331895 |
| State 1 | State 1 | 0.1 | 0.9 |

Table 4. Conditional probability table of the node “SocS”

| SocS1 | SocS2 | SocS4 | SocS5 | Failure Probability | Non-failure Probability |
| --- | --- | --- | --- | --- | --- |
| State 0 | State 0 | State 0 | State 0 | 0.1 | 0.9 |
| State 0 | State 0 | State 0 | State 1 | 0.410195 | 0.589805 |
| State 0 | State 0 | State 1 | State 0 | 0.365753 | 0.634247 |
| State 0 | State 0 | State 1 | State 1 | 0.584353 | 0.415647 |
| State 0 | State 1 | State 0 | State 0 | 0.394277 | 0.605723 |
| State 0 | State 1 | State 0 | State 1 | 0.603046 | 0.396954 |
| State 0 | State 1 | State 1 | State 0 | 0.573136 | 0.426864 |
| State 0 | State 1 | State 1 | State 1 | 0.720259 | 0.279741 |
| State 1 | State 0 | State 0 | State 0 | 0.501068 | 0.498932 |
| State 1 | State 0 | State 0 | State 1 | 0.67303 | 0.32697 |
| State 1 | State 0 | State 1 | State 0 | 0.648393 | 0.351607 |
| State 1 | State 0 | State 1 | State 1 | 0.769578 | 0.230422 |
| State 1 | State 1 | State 0 | State 0 | 0.664206 | 0.335794 |
| State 1 | State 1 | State 0 | State 1 | 0.779941 | 0.220059 |
| State 1 | State 1 | State 1 | State 0 | 0.76336 | 0.23664 |
| State 1 | State 1 | State 1 | State 1 | 0.84492 | 0.15508 |

Table 5. Conditional probability table of the “SocR”

| SocR3 | SocR5 | Failure Probability | Non-failure Probability |
| --- | --- | --- | --- |
| State 0 | State 0 | 0.1 | 0.9 |
| State 0 | State 1 | 0.696648 | 0.303352 |
| State 1 | State 0 | 0.68254 | 0.31746 |
| State 1 | State 1 | 0.892998 | 0.107002 |

Table 6. Conditional probability table of the node “EnvS”

| EnvS1 | EnvS2 | EnvS3 | EnvS4 | Failure Probability | Non-failure Probability |
| --- | --- | --- | --- | --- | --- |
| State 0 | State 0 | State 0 | State 0 | 0.1 | 0.9 |
| State 0 | State 0 | State 0 | State 1 | 0.54408 | 0.45592 |
| State 0 | State 0 | State 1 | State 0 | 0.235279 | 0.764721 |
| State 0 | State 0 | State 1 | State 1 | 0.612609 | 0.387391 |
| State 0 | State 1 | State 0 | State 0 | 0.360512 | 0.639488 |
| State 0 | State 1 | State 0 | State 1 | 0.67605 | 0.32395 |
| State 0 | State 1 | State 1 | State 0 | 0.456633 | 0.543367 |
| State 0 | State 1 | State 1 | State 1 | 0.724743 | 0.275257 |
| State 1 | State 0 | State 0 | State 0 | 0.555276 | 0.444724 |
| State 1 | State 0 | State 0 | State 1 | 0.774713 | 0.225287 |
| State 1 | State 0 | State 1 | State 0 | 0.622122 | 0.377878 |
| State 1 | State 0 | State 1 | State 1 | 0.808576 | 0.191424 |
| State 1 | State 1 | State 0 | State 0 | 0.684005 | 0.315995 |
| State 1 | State 1 | State 0 | State 1 | 0.839924 | 0.160076 |
| State 1 | State 1 | State 1 | State 0 | 0.731502 | 0.268498 |
| State 1 | State 1 | State 1 | State 1 | 0.863985 | 0.136015 |

Table 7. Conditional probability table of the node “EnvR”

| EnvR1 | EnvR2 | EnvR3 | EnvR4 | Failure Probability | Non-failure Probability |
| --- | --- | --- | --- | --- | --- |
| State 0 | State 0 | State 0 | State 0 | 0.1 | 0.9 |
| State 0 | State 0 | State 0 | State 1 | 0.430051 | 0.569949 |
| State 0 | State 0 | State 1 | State 0 | 0.337594 | 0.662406 |
| State 0 | State 0 | State 1 | State 1 | 0.580514 | 0.419486 |
| State 0 | State 1 | State 0 | State 0 | 0.421253 | 0.578747 |
| State 0 | State 1 | State 0 | State 1 | 0.633493 | 0.366507 |
| State 0 | State 1 | State 1 | State 0 | 0.574038 | 0.425962 |
| State 0 | State 1 | State 1 | State 1 | 0.730248 | 0.269752 |
| State 1 | State 0 | State 0 | State 0 | 0.588162 | 0.411838 |
| State 1 | State 0 | State 0 | State 1 | 0.739193 | 0.260807 |
| State 1 | State 0 | State 1 | State 0 | 0.696884 | 0.303116 |
| State 1 | State 0 | State 1 | State 1 | 0.808044 | 0.191956 |
| State 1 | State 1 | State 0 | State 0 | 0.735167 | 0.264833 |
| State 1 | State 1 | State 0 | State 1 | 0.832287 | 0.167713 |
| State 1 | State 1 | State 1 | State 0 | 0.805081 | 0.194919 |
| State 1 | State 1 | State 1 | State 1 | 0.876562 | 0.123438 |

Table 8. Conditional probability table of the node “EcoS”

| EcoS1 | EcoS2 | EcoS3 | EcoS4 | Failure Probability | Non-failure Probability |
| --- | --- | --- | --- | --- | --- |
| State 0 | State 0 | State 0 | State 0 | 0.1 | 0.9 |
| State 0 | State 0 | State 0 | State 1 | 0.353502 | 0.646498 |
| State 0 | State 0 | State 1 | State 0 | 0.459264 | 0.540736 |
| State 0 | State 0 | State 1 | State 1 | 0.611573 | 0.388427 |
| State 0 | State 1 | State 0 | State 0 | 0.566809 | 0.433191 |
| State 0 | State 1 | State 0 | State 1 | 0.688825 | 0.311175 |
| State 0 | State 1 | State 1 | State 0 | 0.739731 | 0.260269 |
| State 0 | State 1 | State 1 | State 1 | 0.813041 | 0.186959 |
| State 1 | State 0 | State 0 | State 0 | 0.322598 | 0.677402 |
| State 1 | State 0 | State 0 | State 1 | 0.513401 | 0.486599 |
| State 1 | State 0 | State 1 | State 0 | 0.593005 | 0.406995 |
| State 1 | State 0 | State 1 | State 1 | 0.707643 | 0.292357 |
| State 1 | State 1 | State 0 | State 0 | 0.673951 | 0.326049 |
| State 1 | State 1 | State 0 | State 1 | 0.765789 | 0.234211 |
| State 1 | State 1 | State 1 | State 0 | 0.804104 | 0.195896 |
| State 1 | State 1 | State 1 | State 1 | 0.859282 | 0.140718 |

Table 9. Conditional probability table of the node “EcoR”

| EcoR1 | EcoR2 | EcoR3 | EcoR4 | Failure Probability | Non-failure Probability |
| --- | --- | --- | --- | --- | --- |
| State 0 | State 0 | State 0 | State 0 | 0.1 | 0.9 |
| State 0 | State 0 | State 0 | State 1 | 0.352674 | 0.647326 |
| State 0 | State 0 | State 1 | State 0 | 0.342277 | 0.657723 |
| State 0 | State 0 | State 1 | State 1 | 0.526932 | 0.473068 |
| State 0 | State 1 | State 0 | State 0 | 0.405252 | 0.594748 |
| State 0 | State 1 | State 0 | State 1 | 0.572227 | 0.427773 |
| State 0 | State 1 | State 1 | State 0 | 0.565356 | 0.434644 |
| State 0 | State 1 | State 1 | State 1 | 0.687382 | 0.312618 |
| State 1 | State 0 | State 0 | State 0 | 0.493051 | 0.506949 |
| State 1 | State 0 | State 0 | State 1 | 0.635376 | 0.364624 |
| State 1 | State 0 | State 1 | State 0 | 0.62952 | 0.37048 |
| State 1 | State 0 | State 1 | State 1 | 0.733532 | 0.266468 |
| State 1 | State 1 | State 0 | State 0 | 0.66499 | 0.33501 |
| State 1 | State 1 | State 0 | State 1 | 0.759045 | 0.240955 |
| State 1 | State 1 | State 1 | State 0 | 0.755175 | 0.244825 |
| State 1 | State 1 | State 1 | State 1 | 0.82391 | 0.17609 |

Table 10. Conditional probability table of the node “Soc”

| SocP | SocS | SocR | Failure Probability | Non-failure Probability |
| --- | --- | --- | --- | --- |
| State 0 | State 0 | State 0 | 0.1 | 0.9 |
| State 0 | State 0 | State 1 | 0.572407 | 0.427593 |
| State 0 | State 1 | State 0 | 0.513808 | 0.486192 |
| State 0 | State 1 | State 1 | 0.769009 | 0.230991 |
| State 1 | State 0 | State 0 | 0.442629 | 0.557371 |
| State 1 | State 0 | State 1 | 0.735191 | 0.264809 |
| State 1 | State 1 | State 0 | 0.698901 | 0.301099 |
| State 1 | State 1 | State 1 | 0.856948 | 0.143052 |

Table 11. Conditional probability table of the node “Env”

| EnvS | EnvR | Failure Probability | Non-failure Probability |
| --- | --- | --- | --- |
| State 0 | State 0 | 0.1 | 0.9 |
| State 0 | State 1 | 0.530407 | 0.469593 |
| State 1 | State 0 | 0.607723 | 0.392277 |
| State 1 | State 1 | 0.795322 | 0.204678 |

Table 12. Conditional probability table of the node “Eco”

| EcoS | EcoR | Failure Probability | Non-failure Probability |
| --- | --- | --- | --- |
| State 0 | State 0 | 0.1 | 0.9 |
| State 0 | State 1 | 0.599032 | 0.400968 |
| State 1 | State 0 | 0.469716 | 0.530284 |
| State 1 | State 1 | 0.763748 | 0.236252 |

Table 13. Conditional probability table of the Urban Community Resilience for PHEs

| Soc | Env | Eco | Failure Probability | Non-failure Probability |
| --- | --- | --- | --- | --- |
| State 0 | State 0 | State 0 | 0.1 | 0.9 |
| State 0 | State 0 | State 1 | 0.421253 | 0.578747 |
| State 0 | State 1 | State 0 | 0.430051 | 0.569949 |
| State 0 | State 1 | State 1 | 0.633493 | 0.366507 |
| State 1 | State 0 | State 0 | 0.566809 | 0.433191 |
| State 1 | State 0 | State 1 | 0.721436 | 0.278564 |
| State 1 | State 1 | State 0 | 0.72567 | 0.27433 |
| State 1 | State 1 | State 1 | 0.823592 | 0.176408 |

Appendix 4

Ranking of influencing factors by PI, CI, and SI.

| Influencing Factor | Probabilistic Importance (PI) | Ranking | Critical Importance (CI) | Ranking | Structural Importance (SI) | Ranking |
| --- | --- | --- | --- | --- | --- | --- |
| Entrance/exit management | 1.94E-02 | 1 | 1.77E-02 | 3 | 1.71E-02 | 3 |
| Medical supplies | 1.90E-02 | 2 | 1.94E-02 | 2 | 1.58E-02 | 6 |
| Population structure | 1.82E-02 | 3 | 1.27E-02 | 8 | 1.97E-02 | 1 |
| Accessibility of medical facilities | 1.77E-02 | 4 | 1.99E-02 | 1 | 1.48E-02 | 7 |
| Sanitation state | 1.77E-02 | 5 | 1.03E-02 | 10 | 1.77E-02 | 2 |
| Social network relationships | 1.55E-02 | 6 | 1.66E-02 | 5 | 1.38E-02 | 8 |
| Level of PHEs | 1.55E-02 | 7 | 1.67E-02 | 4 | 1.67E-02 | 4 |
| Resident income | 1.52E-02 | 8 | 1.48E-02 | 7 | 1.23E-02 | 11 |
| Vulnerable groups | 1.50E-02 | 9 | 1.54E-02 | 6 | 1.64E-02 | 5 |
| Resident health status | 1.33E-02 | 10 | 1.22E-02 | 9 | 1.21E-02 | 12 |
| Rules and regulations | 1.31E-02 | 11 | 7.85E-03 | 13 | 1.29E-02 | 10 |
| Resident belongingness | 1.26E-02 | 12 | 6.64E-03 | 15 | 1.34E-02 | 9 |
| Capital investment | 1.17E-02 | 13 | 4.71E-03 | 19 | 1.15E-02 | 13 |
| Intelligent supervision | 1.05E-02 | 14 | 9.15E-03 | 11 | 9.22E-03 | 14 |
| Community quality | 9.75E-03 | 15 | 8.29E-03 | 12 | 8.84E-03 | 16 |
| Communication system | 9.22E-03 | 16 | 4.05E-03 | 22 | 8.78E-03 | 17 |
| Social insurance | 9.17E-03 | 17 | 4.30E-03 | 21 | 8.77E-03 | 18 |
| Transportation robustness | 8.77E-03 | 18 | 5.44E-03 | 17 | 8.90E-03 | 15 |
| Emergency shelter | 8.62E-03 | 19 | 5.85E-03 | 16 | 8.62E-03 | 19 |
| Public service | 7.69E-03 | 20 | 7.31E-03 | 14 | 7.02E-03 | 20 |
| Past experience | 6.65E-03 | 21 | 5.35E-03 | 18 | 6.76E-03 | 21 |
| Community assets | 6.48E-03 | 22 | 4.34E-03 | 20 | 5.77E-03 | 23 |
| Living supporting facilities | 5.95E-03 | 23 | 3.18E-03 | 23 | 6.03E-03 | 22 |
| Resident employment | 5.50E-03 | 24 | 3.12E-03 | 24 | 4.96E-03 | 25 |
| Government leadership functions | 5.08E-03 | 25 | 2.16E-03 | 26 | 5.54E-03 | 24 |
| Public space | 4.51E-03 | 26 | 2.26E-03 | 25 | 4.24E-03 | 26 |
| Publicity and education | 3.02E-03 | 27 | 2.07E-03 | 27 | 3.33E-03 | 27 |
| Resident educational level | 2.70E-03 | 28 | 1.91E-03 | 28 | 1.78E-03 | 28 |

Appendix 5

Definition and measurement of factors influencing urban community resilience for PHEs

| Identification Dimension | Influencing Factors | Definition | Measurement Criteria |
| --- | --- | --- | --- |
| Environmental Resilience State  (EnvS) | Sanitation state  (EnvS1) | Implement waste management, regular sanitation of public facilities, and thorough area disinfection to maintain a hygienic environment and curb the spread of viruses and diseases. | Residents' satisfaction with waste management and sanitation services, as an indicator of community hygiene and public health. |
|  | Community quality  (EnvS2) | Planning factors including residential distribution, sunlight exposure, ventilation, greening levels, and building types collectively influence the comfort and sanitary conditions of residents. | The community floor area ratio, green space ratio, and levels of sunlight and ventilation. |
|  | Public space  (EnvS3) | Outdoor public spaces—such as parks, green areas, plazas, and public facilities—create ventilation corridors that enhance air quality, suppress viral spread, and promote physical and mental well-being. | The per capita public space area within the community, as well as the uniform accessibility and sanitary safety of internal and adjacent green spaces, plazas, and parks. |
|  | Entrance/exit management  (EnvS4) | During emergency lockdowns, community access points—incorporating temperature screening, registration, and logistical functions—act as the first line of defense to protect residents' health and safety. | The rationality of community entrance and exit planning, its effectiveness in epidemic prevention, and the feasibility of implementing closed-off management. |
| Environmental Resilience Response  (EnvR) | Accessibility of medical facilities (EnvR1) | Residents should have timely and convenient access to medical services when required, supported by the community’s capacity to provide healthcare resources and maintain sanitation protocols. | The number and scale of medical facilities within a 2km radius, addressing residents' healthcare needs during both routine and epidemic periods. |
|  | Emergency shelter (EnvR2) | Flexible community facilities that can adapt to varying needs during different phases. For instance, communities may be repurposed as isolation centers during outbreaks. | The number of venues, hotels, and multi-purpose activity centers within a 15-30 minute daily living radius. |
|  | Living supporting facilities  (EnvR3) | Essential infrastructure that supports residents' daily needs and safeguards quality of life during epidemics, ensuring the convenience of daily living. | The availability of supermarkets, pharmacies, parcel stations, cultural and sports facilities, and community service stations within a 15-minute daily living radius. |
|  | Transportation robustness  (EnvR4) | Transportation networks within and outside communities must maintain social distancing during crises, facilitating efficient emergency responses, personnel evacuations, and supply deliveries. | The number of bus and subway stops within a 1km radius, and whether community roads are level, clean, and meet width requirements. |
| Economic Resilience State  (EcoS) | Resident employment (EcoS1) | The employment status of legal working-age residents engaged in remunerative activities influences their capacity to respond to PHEs. | The employment rate and occupational distribution of community residents. |
|  | Resident income (EcoS2) | All economic income from residents' employment and household businesses, including wages, self-employment income, savings, and other sources, reflects their economic stability and resilience to disasters. | The per capita monthly disposable income of community residents in comparison to the city average. |
|  | Social insurance (EcoS3) | Community social insurance coverage, combined with residents’ purchases of financial products like health and accident insurance, strengthens the economic security of individuals and households against risks. | The proportion of residents covered by social security or other forms of insurance. |
|  | Community assets (EcoS4) | Property rights to real estate owned by individuals within the community, including housing and commercial premises, as well as the diversity, scale, and attractiveness of business formats within the community's economic framework. | The proportion of residents owning high-value fixed assets, such as vehicles or real estate, as well as community shop rental rates and the commercial floor area ratio. |
| Economic Resilience Response  (EcoR) | Medical supplies  (EcoR1) | The community routinely prepares essential epidemic prevention and living supplies and allocates them efficiently during emergencies or ongoing health crises. | Whether residents experienced shortages of essential supplies during the pandemic period. |
|  | Capital investment  (EcoR2) | The comprehensive process of mobilizing, planning, utilizing, and overseeing community-level epidemic prevention funds through multiple channels during public health crises. | Whether the community has adequate epidemic prevention funds or dedicated budgets for disaster prevention and mitigation. |
|  | Communication system (EcoR3) | The community utilizes mobile social tools and online platforms to update, disseminate, and transmit epidemic prevention policies and information, while promoting public health knowledge. | The availability of online communication platforms between the community and property owner associations. |
|  | Intelligent supervision (EcoR4) | Development of an intelligent epidemic prevention system using big data and network technology for real-time monitoring, risk early warnings, and enabling smart security and data sharing. | The presence of smart epidemic prevention facilities capable of real-time public health monitoring and issuing risk alerts. |
| Social resilience Pressure  (SocP) | Level of PHEs  (SocP1) | PHEs are categorized into different alert levels based on their cause, severity, scope, and spread trajectory. | The classification is primarily based on alert levels, with reference to cumulative confirmed cases and the incidence rate per million people. |
|  | Vulnerable groups  (SocP2) | Populations vulnerable to PHEs due to frailty, limited resources, or other factors, including the elderly, children, disabled individuals, and unemployed welfare recipients. | Whether vulnerable groups constitute more than 20% of the total community population and whether community safeguards are in place. |
| Social resilience State  (SocS) | Population structure (SocS1) | Compositional characteristics of the community population, including factors such as population mobility, density, and age distribution. | The number of non-local residents, population density, and age distribution within the community. |
|  | Resident health status (SocS2) | The physical and mental health status of community residents, with good health manifesting as enhanced physical fitness, sustained immunity, and a positive mindset. | Health status categorized into five levels: diseased, sub-health, fair, good, and healthy. |
|  | Resident educational level (SocS3) | The overall educational attainment and distribution of highest academic qualifications among community residents. | The percentage of residents aged 15 and above holding college degrees or higher. |
|  | Risk awareness  (SocS4) | Residents' attitudes toward recognizing, preventing, and responding to PHEs, such as infectious diseases, including stockpiling essential supplies, adopting protective measures, and complying with response policies. | Residents' awareness of and preparedness for PHEs. |
|  | Rules and regulations (SocS5) | Norms and guiding principles—such as contracts, cooperation, and integrity—that effectively regulate resident behavior and maintain community order, including public health regulations and community emergency response protocols. | The existence of community emergency response plans for public health incidents and residents' familiarity with relevant community regulations. |
|  | Publicity and education  (SocS6) | Educational campaigns to enhance residents' knowledge and capabilities in epidemic prevention and disaster preparedness, sharing community experiences. | The conduct of public health awareness campaigns, training programs, and educational activities. |
| Social resilience Response (SocR) | Resident belongingness (SocR1) | Community members' subjective reliance on and positive evaluation of the community, including trust in community workers and identification with community culture. This cohesion aids and strengthens collaborative community prevention and control capabilities. | The strength of residents' emotional attachment to the community, including trust, sense of belonging, shared values, and community culture. |
|  | Public service  (SocR2) | Property management and community services provided by self-governance organizations or volunteers to safeguard residents' health and meet basic living needs. | Resident satisfaction with community services, including property management and Party-mass organizations. |
|  | Public participation (SocR3) | The participation level of community residents and social forces in epidemic prevention and disaster mitigation, including volunteer assistance, social organization cooperation, and oversight of control measures.. | Residents' willingness and level of cooperation in participating in volunteer organizations and community epidemic prevention efforts. |
|  | Social network relationships  (SocR4) | Interactions among community entities, including neighborhood connections and collaborations on shared goals, such as epidemic prevention and control. | The frequency of interaction and mutual assistance among community members and between the community and external entities. |
|  | Community emergency management capability (SocR5) | The ability to coordinate resources effectively in response to PHEs, establish professional epidemic prevention structures, and plan and execute management measures. | Whether the community promptly established an epidemic prevention emergency team with clearly defined responsibilities. |
|  | Past experience  (SocR6) | The ability to analyze the causes and progression of PHEs, learn from communication, reflection, and process improvement, and adapt to future crises. | Whether the community implemented targeted improvement projects following the epidemic. |
|  | Government leadership functions  (SocR7) | The capacity of governments and grassroots Party-government agencies to lead during PHEs, ensure efficient decision-making, and organize prevention and control efforts. | The intensity of guidance, management deployment, and personnel support provided by government or grassroots agencies for urban and community epidemic prevention actions, ranked from weakest to strongest. |
